# Two-Dimensional Moiré Phonon Polaritons

**DOI:** 10.1021/acs.nanolett.5c03046

**Published:** 2025-10-16

**Authors:** Hao Shi, Chu Li, Ding Pan, Xi Dai

**Affiliations:** † New Cornerstone Science Laboratory, Department of Physics, 58207 The Hong Kong University of Science and Technology, Hong Kong, China; ‡ Department of Physics, The Hong Kong University of Science and Technology, Hong Kong, China; § Department of Chemistry, The Hong Kong University of Science and Technology, Hong Kong, China

**Keywords:** phonon polariton, two-dimensional material, moiré superlattice, near-field optics, spectral reconstruction

## Abstract

Phonon polaritons (PhPs) are hybrid light–matter modes. We investigate them in two-dimensional (2D) materials with twisted moiré structures, revealing that the moiré potential creates a new class of “moiré PhPs”. These exhibit a fundamental spectral reconstruction into multiple branches and, crucially, electromagnetic wave functions that are nanopatterned by the superlattice. Through numerical simulations based on realistic lattice models, we confirm the existence of these intriguing modes. The inherent nanoscale structuring produces a robust, spatially varying near-field response, establishing moiré superlattices as a platform for engineering light–matter interactions.

## Graphical abstract

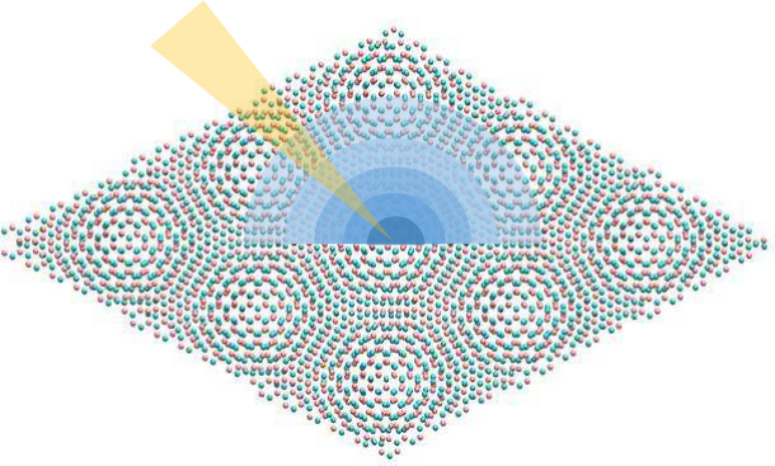

Polaritons arise from the coupling of photons with collective excitations in materials such as phonons, plasmons, and excitons. These hybrid modes exhibit properties of both light and matter, enabling broad applications in fields like optics,^1,2^ condensed matter physics,^3,4^ and quantum computing.^5,6^ In polar crystals, ions oscillate with polarization and interact with electromagnetic (EM) waves. The coupling between ionic motion and the EM field produces phonon polaritons (PhPs). The first PhP model for 3D crystals was established by Huang’s equations,^7,8^ which treat long-wavelength ionic vibrations and polarization macroscopically. Solving Huang’s equations alongside Maxwell’s equations yields 3D PhPs. A similar macroscopic theory can also be applied to 2D materials, though it incorporates additional constraints from EM boundary conditions.^9–17^ In 2D systems, a PhP can manifest as a transverse magnetic (TM) or transverse electric (TE) mode, propagating along the material surface.

Moiré superlattices offer a novel approach to engineer 2D physics at length scales far exceeding the crystal periodicity, serving as a powerful platform for light–matter interactions.^4,18–20^ The discovery of superconducting and correlated insulating states in twisted bilayer graphene^21,22^ has spurred the observation of exotic phenomena in moiré systems.^18,19,23–35^ Despite widespread interest and progress, PhPs in moiré systems remain poorly explored, likely due to the limited optical resolution of the tiny energy scales characteristic of moiré physics. Previous work has explored PhPs primarily in thicker twisted structures where modulation of polariton propagation dominates.^36–42^ However, a study focused on atomically thin layers is missing. Additionally, experimental samples often exhibit high dissipation, complicating direct detection of moiré polaritons. Theoretically, the challenge lies in managing the vast degrees of freedom inherent to moiré superlattices.

In this study, we investigate PhPs in moiré materials, specifically, twisted bilayer hexagonal boron nitride (hBN) and MoTe_2_, using lattice models. We reveal that the moiré potential gives rise to a new class of PhPs with two defining characteristics: (I) a fundamental spectral reconstruction into multiple, flat PhP branches (Figure 2) and (II) most importantly, electromagnetic wave functions that are nanopatterned by the moiré lattice itself (Figure 3). Unlike the multiple branches in slabs that arise from anisotropy or vertical mode hybridization,^4,12,43,44^ the multiple flat bands in moiré PhPs emerge from moiré-driven mini-band formation and zone-folding effects. This results in a unique physical phenomenon: long-wavelength evanescent light can excite confined optical states with spatial features orders of magnitude smaller than the photon’s wavelength—a form of inherent nanoscale optical structuring absent in conventional materials. This manifests as a spatially inhomogeneous local response^18,19^ that provides a robust, experimentally accessible signature via near-field techniques (Figure 4),^41^ even when the fine spectral details are obscured by a realistic phonon line width. Moiré PhPs establish a unique nanophotonic platform that merges twist-tunability with phonon-based mid-infrared confinement, enabling programmable nanoscale optical fields distinct from both conventional polar dielectrics and electron-based moiré systems.^4,45^

**Figure 1:**
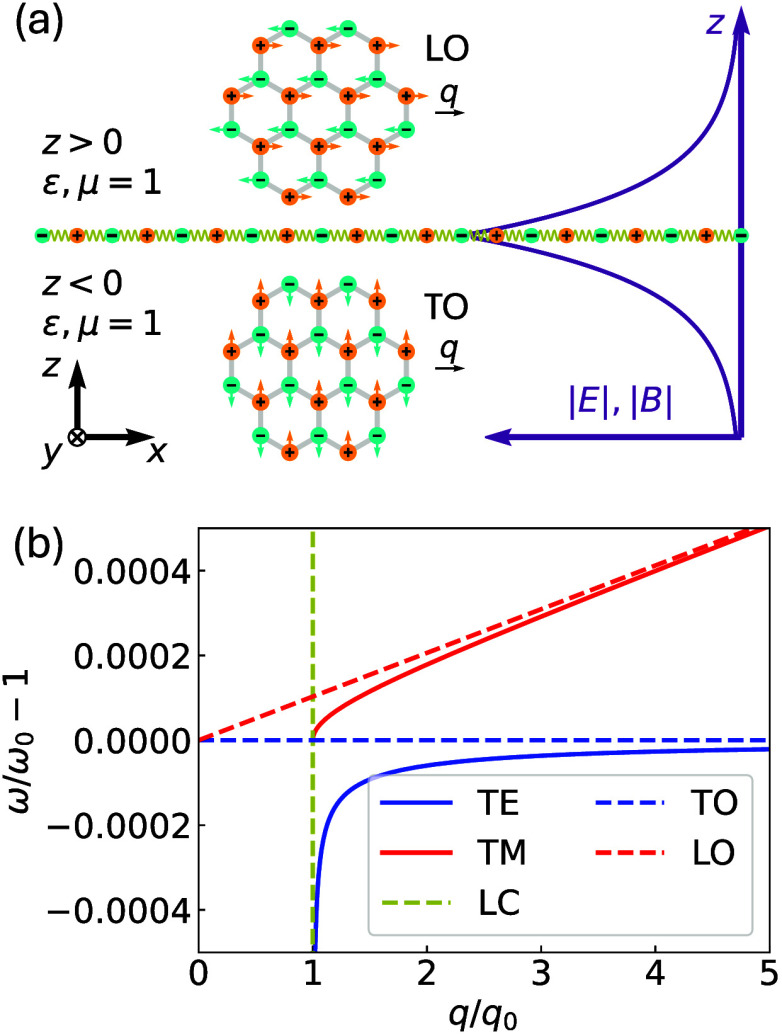
(a) A 2D polar sheet is positioned at *z* = 0 in a vacuum. The PhP exhibits characteristic 2D EM waves that decay along the *z*-axis, as illustrated by the purple coordinate system. The inset displays the long-wavelength (***q*** = *q* ***e***_*x*_) LO and TO mode patterns in the *xy*-plane for a binary crystal. (b) The 2D PhP dispersion of the TM and TE modes near the light cone (LC) and resonance frequency ω_0_. For comparison, the LO and TO modes under the nonretarded approximation are also shown. In (b), we use *T*/(2ω_0_ *c*) = 2.06 × 10^–4^, obtained from the lattice model of monolayer hBN (SI Section 2.2).

**Figure 2:**
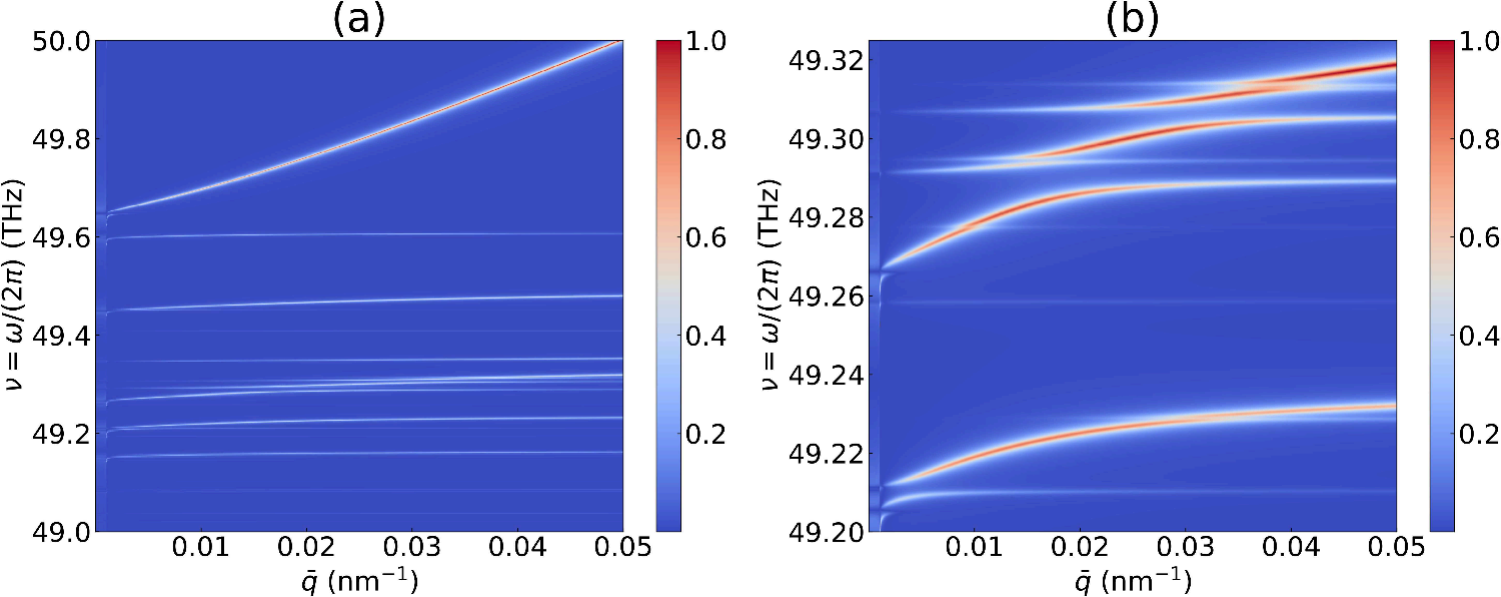
(a) The long-wavelength PhP dispersion of 2.65° twisted bilayer hBN near ν_0_ = ω_0_/(2π) ≈ 49.5 THz (*q*_0_ ≈ 10^–3^ nm^–1^), along the Γ̅−M̅ line, obtained by plotting the (normalized) spectrum ln(1+|L(q̅,ω)|) . Here a tiny line width δ/(2π) = 10^–3^ THz is used to make each branch distinguishable. Many flat branches appear below the topmost dominant branch. (b) The detailed dispersion within the mini-window 49.20–49.325 THz.

**Figure 3:**
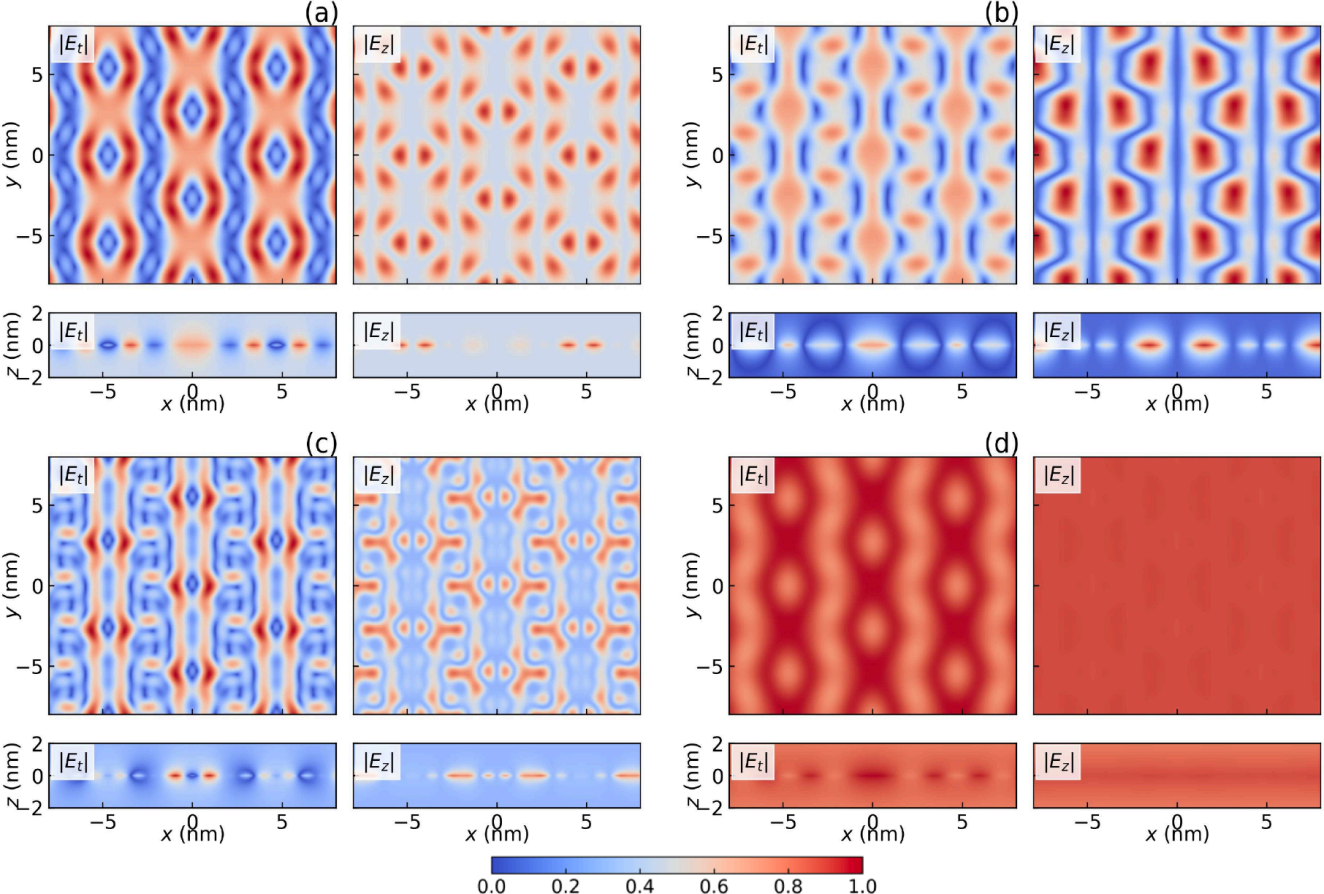
Field distributions of moiré PhPs in 2.65° twisted bilayer hBN: in-plane (*z* = 0, top row) and out-of-plane (*y* = 0, bottom row) amplitudes | ***E***_*t*_| and |*E*_*z*_| along the Γ̅−M̅ line at (a) $\mathrm{q̅}=0.01\;{\mathrm{nm}}^{-1}$ , ν = 49.696 THz; (b) $\mathrm{q̅}=0.01\;{\mathrm{nm}}^{-1}$ , ν = 49.602 THz; (c) $\mathrm{q̅}=0.01\;{\mathrm{nm}}^{-1}$ , ν = 49.217 THz; (d) $\mathrm{q̅}=0.05\;{\mathrm{nm}}^{-1}$ , ν = 50.002 THz. Fields are normalized to maxima of 1. (a), (b), and (c) indicate that, at a fixed q̅ , the specific moiré pattern of EM waves is sensitive to the frequency. (a) and (d) are taken from the same branch.

**Figure 4:**
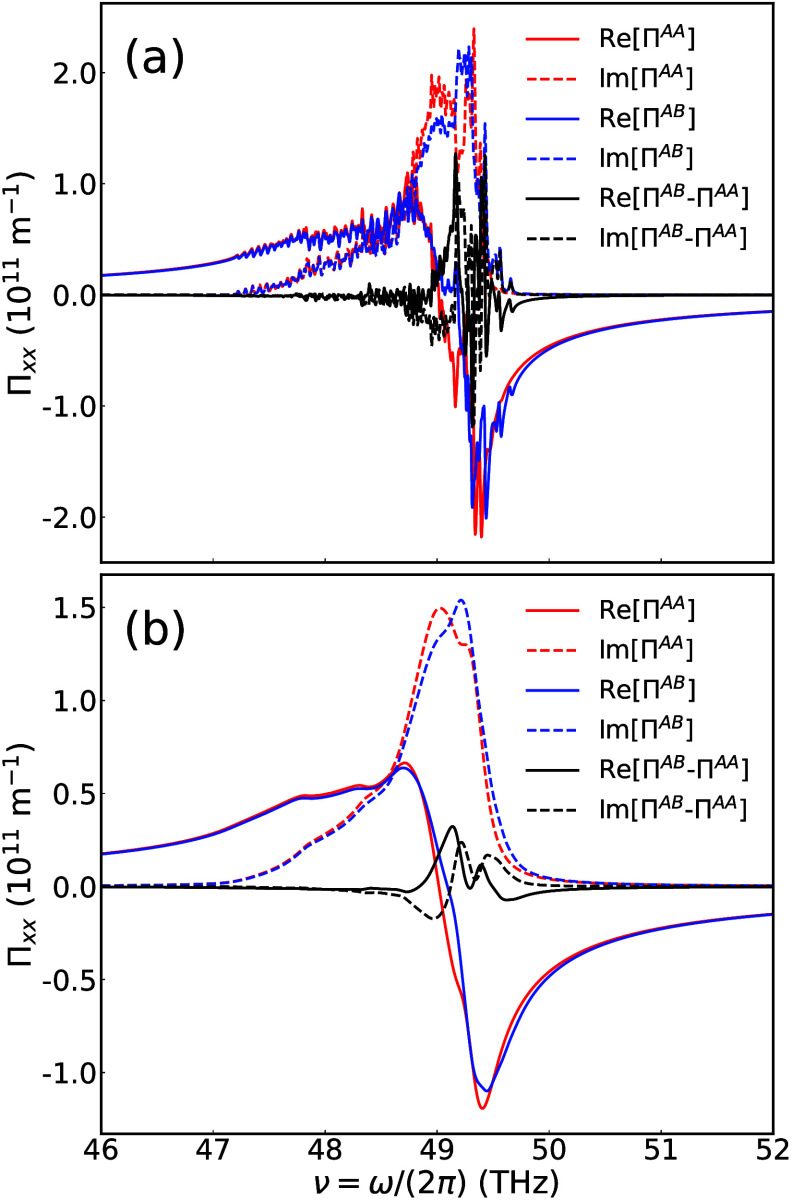
Local susceptibility as a function of frequency, calculated using line widths (a) δ/(2π) = 0.015 THz (0.5 cm^–1^) and (b) δ/(2π) = 0.15 THz (5 cm^–1^). The red and blue curves denote the values at AA and AB points. The solid and dashed lines represent the real and imaginary parts. The black lines indicate their difference.

We begin with the 2D PhP formalism. Consider an ionic sheet positioned at *z* = 0 in a vacuum [ϵ, μ = 1, Figure 1(a)]. Its dynamics are governed by the vibration field ***W*** describing the in-plane ionic motion, which obeys the equation of motion:

1 $$Ẅ=-\omega_{0}^{2}W+\gamma_{12}E_{t}$$

where ω_0_ is the resonance frequency and ***E***_*t*_ denotes the in-plane component of the electric field ***E*** at *z* = 0. The in-plane polarization density ***P*** arises primarily from ionic displacement,

2 $$P=\gamma_{21}W$$

Here, $\gamma_{12}=\gamma_{21}=\sqrt{\varepsilon_{0}T}$ can be derived from microscopic models. These equations represent the 2D analogs of Huang’s equations and must be solved together with Maxwell’s equations and the boundary conditions at *z* = 0. We seek solutions of the form ***E***, ***W*** ∝ *e* ^*i* ***q*** · ***r***–*iωt*^, where ***r*** and ***q*** are the *in-plane* position and momentum, respectively. The susceptibility is then defined as

3 $$P=\varepsilon_{0}\Pi \left(\omega \right)E_{t},,\Pi \left(\omega \right)=\frac{T}{\omega_{0}^{2}-\omega^{2}}$$

The above equations have guided or radiative solutions,^46,47^ depending on whether the decaying parameter $\lambda =\sqrt{q^{2}-\omega^{2}/c^{2}}$ is real or imaginary. Radiative modes correspond to conventional light propagation problems with the polar sheet acting as a scattering interface [Supporting Information (SI) Section 1.4]. Our focus, however, is on guided modes that feature localized 2D EM waves near *z* = 0.^48^ The guided modes split into an *s*-polarized (TE) mode with ***E*** ⊥ ***q*** and a *p*-polarized (TM) mode with ***B*** ⊥ ***q***. The dispersions of the TE and TM modes are shown in Figure 1(b), which are governed by the eigen equations 1 – Π­(ω)­ω^2^/(2*λc* ^2^) = 0 and 1 + λΠ­(ω)/2 = 0, respectively (SI Section 1.3). The TE mode resembles free-space light at *q* ≪ ω_0_/*c*, while it converges to pure lattice oscillations at *q* ≫ ω_0_/*c*. The TM mode’s (long-wavelength) dispersion starts at ω_0_ = *cq*_0_ and tends to linear dispersion at *q* ≫ ω_0_/*c*. They are quintessential 2D EM waves with a power density localized along *z*, arising universally in 2D materials and 3D material interfaces due to polarizable collective modes. The conditions to determine the eigenmodes are quite general: for example, substituting Π­(ω) with its plasmonic counterpart extends the framework to 2D plasmon polaritons. Critically, the TM (TE) mode requires Π­(ω) < 0 [Π­(ω) > 0]. This sign rule for polarization persists; for instance, graphene’s interband conductivity enables Π­(ω) < 0 in a specific regime, hosting a unique TE plasmon mode^49,50^ absent in conventional 2D electron gas.^51^

It is instructive to consider the nonretarded limit (*q* ≫ ω/*c*), where retardation effects are neglected and the Coulomb interaction is treated as instantaneous. In this limit, the TM and TE modes reduce to the transverse optical (TO) and longitudinal optical (LO) phonon modes, respectively (SI Section 1.2). Their dispersions are shown as dashed lines in Figure 1(b). The TO mode corresponds to a pure mechanical oscillation where ***E*** = **0** and ***W*** ⊥ ***q*** vibrates at a fixed frequency ω_TO_ = ω_0_. In contrast, the LO mode involves a macroscopic ***E*** field that couples to the vibration. Its dispersion is governed by 1 + *q*Π­(ω)/2 = 0. From this, a characteristic linear LO–TO splitting can be derived in the long-wavelength limit: ω_LO_ – ω_TO_ ≈ *qT*/(4ω_0_). This linear splitting is a fundamental signature of 2D polar systems,^11,12,14,16^ arising from the long-range Coulomb interaction in a reduced dimension. It stands in stark contrast to the behavior in 3D bulk crystals, where the large depolarizing field leads to a *q*-independent splitting at the Brillouin zone center.^7,8^ This key difference highlights the profound effect of dimensionality on light–matter interactions in polar materials.

Both guided and radiative modes can also be treated within a unified framework of light reflection and refraction (SI Section 1.5). In this approach, the PhP dispersion ω­(***q***) emerges as the poles of the transmission matrix *T*(***q***, ω), offering computational advantages.^52^ The spectrum can be visualized by plotting L(q,ω)=−Im[det[T(q,ω+iδ/2)]] , where δ (representing the phonon line width) is tiny. This method simultaneously captures the continuous spectrum of radiative solutions and discrete dispersions of guided modes.

The physics becomes richer in moiré systems, where the supercell can reach mesoscopic scales with vast sublattice degrees of freedom.^41^ Phonons fold into the moiré Brillouin zone (mBZ), generating intricate moiré phonon bands.^53,54^ This raises a compelling question: how do PhPs emerge in such complex systems amid long-range EM interactions? For quantitative analysis, we utilize realistic lattice models that bypass computationally intense *ab initio* methods.^55^ Short-range ionic interactions are modeled via a force field (SI Section 6), while long-range Coulomb forces are treated through macroscopic electric fields. The displacement ***u*** of an ion at position ***r***_*Iiα*_ (where *I*, *i*, and α index the supercell, atomic cell, and sublattice positions, respectively, as detailed in SI Section 3.1) satisfies the following equation of motion:

4 $$\begin{array}{l} M_{\alpha }ü\left(r_{Ii\alpha }\right)+\underset{Jj\beta }{\sum }\Phi_{i\alpha ,j\beta }\left(r_{Ii\alpha }-r_{Jj\beta }\right)u\left(r_{Jj\beta }\right)\\ -\underset{Q}{\sum }Z_{\alpha }eE_{\mathrm{q̅}+Q,t}e^{i\left(\mathrm{q̅}+Q\right)\cdot r_{Ii\alpha }-i\omega t}=0 \end{array}$$

where Φ is the force constant^56–64^ and *M*_α_ and *Z*_α_ are the ionic mass and charge (in units of *e*), respectively. The moiré electric field $E_{t}=\underset{Q}{\sum }E_{\mathrm{q̅}+Q,t}e^{i\left(\mathrm{q̅}+Q\right)\cdot r-i\omega t}$ includes components indexed by moiré reciprocal vectors ***Q***, with q̅∈ mBZ. The final term in eq 4 is the driving force from the macroscopic electric field, which encodes the long-range 2D Coulomb interaction essential for PhP formation. The polarization density is given by^8,12,65,66^

5 $$P\left(r\right)=\underset{Ii\alpha }{\sum }Z_{\alpha }eu\left(r_{Ii\alpha }\right)\delta \left(r-r_{Ii\alpha }\right)$$

These equations generalize eqs 1 and 2 to the lattice level.^67^ Without ***E***_*t*_, eq 4 reduces to the standard nonpolar phonon problem. The driven harmonic oscillator system admits an exact solution,^8^ yielding a susceptibility tensor with multiple poles due to the moiré potential (SI Section 3). In Fourier basis, $P\left(r\right)=\underset{Q}{\sum }P_{\mathrm{q̅}+Q}e^{i\left(\mathrm{q̅}+Q\right)\cdot r-i\omega t}$ , we obtain

6 $$\begin{array}{ll} & P_{\mathrm{q̅}+Q}=\varepsilon_{0}\underset{Q^{\prime }}{\sum }\Pi^{QQ^{\prime }}\left(\mathrm{q̅},\omega \right)E_{\mathrm{q̅}+Q^{\prime },t},\\ & \Pi^{QQ^{\prime }}\left(\mathrm{q̅},\omega \right)=\frac{e^{2}}{\varepsilon_{0}\Omega_{m}}\underset{b}{\sum }\frac{S_{Qb}\left(\mathrm{q̅}\right)S_{Q^{\prime }b}^{†}\left(\mathrm{q̅}\right)}{\omega_{\mathrm{q̅}b}^{2}-\omega^{2}} \end{array}$$

where *ε*_0_ is the vacuum permittivity, Ω_*m*_ is the supercell area, $\omega_{\mathrm{q̅}b}$ and $e_{b}\left(\mathrm{q̅}\right)$ the bare frequency and displacement vector of the *b*-th moiré phonon without ***E***_*t*_, and the *S* matrix is

7 $$S_{Qb}\left(\mathrm{q̅}\right)=\underset{i\alpha }{\sum }\frac{Z_{\alpha }e_{i\alpha ,b}\left(\mathrm{q̅}\right)}{\sqrt{M_{\alpha }}}e^{-iQ\cdot \left(R_{i}+\tau_{\alpha }\right)}$$

The moiré physics manifests in the off-diagonal terms of **Π** ^***QQ***^′. The ***Q*** ≠ **0** terms encode field modulations at moiré length scales.^18^ If we turn off the moiré potential, eq 6 becomes diagonal in ***Q***, recovering the moiré-free case (SI Section 5.4).

The moiré PhPs are determined by solving Maxwell’s equations with appropriate boundary conditions. Assuming an infinitesimally thin moiré material for simplicity, the eigenmode problem reduces to solving the secular equation det[A(q̅,ω)]=0 , where A(q̅,ω) is a block-structured matrix acting on the space of ***Q***, encoding the material’s light-scattering properties (SI Section 3.2). The matrix elements are

8 $$\begin{array}{ll} & A_{∥∥}^{QQ^{\prime }}\left(\mathrm{q̅},\omega \right)=\delta_{QQ^{\prime }}+\frac{\lambda_{\mathrm{q̅}+Q}}{2}\Pi_{∥∥}^{QQ^{\prime }}\left(\mathrm{q̅},\omega \right),\\ & A_{∥⊥}^{QQ^{\prime }}\left(\mathrm{q̅},\omega \right)=\frac{\lambda_{\mathrm{q̅}+Q}}{2}\Pi_{∥⊥}^{QQ^{\prime }}\left(\mathrm{q̅},\omega \right),\\ & A_{⊥∥}^{QQ^{\prime }}\left(\mathrm{q̅},\omega \right)=-\frac{1}{2\lambda_{\mathrm{q̅}+Q}}\frac{\omega^{2}}{c^{2}}\Pi_{⊥∥}^{QQ^{\prime }}\left(\mathrm{q̅},\omega \right),\\ & A_{⊥⊥}^{QQ^{\prime }}\left(\mathrm{q̅},\omega \right)=\delta_{QQ^{\prime }}-\frac{1}{2\lambda_{\mathrm{q̅}+Q}}\frac{\omega^{2}}{c^{2}}\Pi_{⊥⊥}^{QQ^{\prime }}\left(\mathrm{q̅},\omega \right) \end{array}$$

Here, ∥ and ⊥ denote components parallel and perpendicular to $\mathrm{q̅}+Q\left(Q^{\prime }\right)$ , respectively, with $\lambda_{\mathrm{q̅}+Q}=\sqrt{|\mathrm{q̅}+Q{|}^{2}-\omega^{2}/c^{2}}$ . Equation 8 is the central result of our work, which contains all the information about moiré PhPs. The transmission matrix can be obtained from the *A* matrix as $T\left(\mathrm{q̅},\omega \right)=A^{-1}\left(\mathrm{q̅},\omega \right)$ . The PhP dispersion can be obtained by searching the zeros of det­(*A*) [poles of det­(*T*)], and the corresponding eigenmodes can be obtained as null vectors of *A*. A key feature of moiré PhPs is that an incident evanescent wave (with long in-plane wavelength) can excite EM fields with much shorter wavelengths. This occurs through moiré potential scattering, which is encoded in the off-diagonal elements (in ***Q***) of the scattering matrix A(q̅,ω) (SI Section 3.2). So we focus exclusively on the case where the incident light has ***Q*** = **0** components only. The effective transmission matrix is the long-wavelength block of the full transmission matrix $T_{\mathrm{eff}}\left(\mathrm{q̅},\omega \right)={\left[A^{-1}\left(\mathrm{q̅},\omega \right)\right]}^{00}$ .^19,32,68^ The poles of the spectrum L(q̅,ω)=−Im[det[Teff(q̅,ω+iδ/2)]] depict the dispersion of moiré PhPs that can be excited by long-wavelength light.

We select hBN and MoTe_2_ as two examples, which are popular insulating polar crystals.^27,28,41,69,70^ Our analysis focuses on AA-stacked twisted bilayer configurations of these materials. A different stacking style could slightly influence the PhP dispersion but would not alter the moiré physics discussed here. While our numerical examples focus on hexagonal lattices, the above formalism is general and applicable to any 2D moiré polar system.

Hexagonal boron nitride is a prototypical polar material for PhP studies,^12–16,71^ featuring an optical phonon frequency ν_0_ = ω_0_/(2π) ≈ 49.4 THz from our molecular dynamics-based lattice model. While this exceeds the experimental value of ∼41 THz, the necessity of our model for capturing the full moiré potential is worth the cost of accuracy for the frequency. We adopt isotropic charges *Z*_B_ = −*Z*_N_ ≈ 2.7 ^14^ and focus on 2.65° twisted bilayer hBN that has lattice length *L*_θ_ ≈ 5.42 nm and 1876 atoms per supercell. The long-wavelength dispersion near ω_0_ is shown in Figure 2, where many PhP branches appear. Although the phonon moiré potential is weak in magnitude, it effectively hybridizes the long-wavelength (***Q*** = **0**) components with shorter-wavelength (***Q*** ≠ **0**) components through non-negligible off-diagonal terms in the susceptibility tensor eq 6, particularly near the resonance frequency $\omega_{\mathrm{q̅}b}$ . This hybridization generates new PhP branches exhibiting characteristic moiré interference patterns. The resulting dispersions exhibit sharp transitions between spectral regions bounded by folded phonon frequencies $\omega_{\mathrm{q̅}b}$ , forming a series of mini-bands in the polariton spectrum. The dominant branch above 49.6 THz resembles the TM mode without moiré potential. The neighboring phonon frequencies $\omega_{\mathrm{q̅}b}$ stay very close to each other. Therefore, the emerging moiré PhP modes are quite flat, with energy resolutions on the scale of ∼0.01 THz [Figure 2(b)]. This fine structure would be significantly obscured under a more realistic line width δ ^72–74^ (SI Section 3.3). Consequently, resolving the full moiré PhP dispersion poses a significant experimental challenge and requires samples with exceptionally low dissipation. All eigenmodes represent genuine moiré PhPs, as their EM fields (and lattice oscillations) exhibit varying degrees of wavelength mixing. The electric fields ***E*** for some representative modes are plotted in Figure 3. Nanoscale modulations of ***E*** are clearly observed in the *xy*-plane, where each PhP branch exhibits a unique moiré pattern that precisely follows the moiré superlattice periodicity. These patterns are highly sensitive to both frequency [Figure 3(a)–(c)] and momentum q̅ [Figure 3(a) vs (d)], enabling programmable optical hotspots that can be spatially reconfigured through excitation tuning. Meanwhile, the lower panels of Figure 3 illustrate the field distribution along the *z*-axis, showing that the moiré pattern is confined within the material layer (on the scale of *L*_θ_) while the long-wavelength component of the field extends further. These characteristic spatial signatures of moiré PhPs are absent in moiré-free systems.

Another key feature of moiré polar systems is their spatially varying local response. This provides important signatures for detection using scanning near-field optical microscopy (SNOM).^41,75^ In SNOM measurements, a tightly focused light field ***E*** ∼ δ­(***r*** – ***r***_0_)*e* ^–*iωt*^ illuminates the sample, and the response at the same position ***r***_0_ is measured. This technique probes the local susceptibility **Π**(***r***, ***r***, ω), which in our formalism can be calculated as (*N*_*m*_Ω_*m*_ is the sample area):

9 $$\Pi \left(r,r,\omega \right)=\frac{1}{N_{m}\Omega_{m}}\underset{\mathrm{q̅}QQ^{\prime }}{\sum }\Pi^{QQ^{\prime }}\left(\mathrm{q̅},\omega \right)e^{i\left(Q-Q^{\prime }\right)\cdot r}$$

We see that a system can have an inhomogeneous local response; that is, **Π**(***r***, ***r***, ω) depends explicitly on ***r***, if and only if $\Pi^{QQ^{\prime }}\left(\mathrm{q̅},\omega \right)$ is not diagonal about ***Q***. This rules out the possibility of observing spatially varying signals in moiré-free systems such as monolayer hBN. We numerically calculate eq 9 at two different stacking points, AA and AB, using a 7 × 7 sample mesh of q̅ , 61 truncated ***Q*** vectors, and two different phonon line widths δ. The results of Π_*xx*_ in the frequency window 46–52 THz are shown in Figure 4 (time reversal and *C*_3*z*_ symmetries require Π to be proportional to the identity matrix, as shown in SI Section 5.3). In Figure 4(a), using a tiny δ leads to the sawtooth pattern of Π_*xx*_. Each peak corresponds to a specific moiré mode. These sharp features are smeared when a larger, more realistic δ is used, as shown in Figure 4(b). The signal difference between the AA and AB points becomes pronounced in a narrower window (48.5–50 THz), where moiré PhPs are active [Figure 2(a)]. Outside this range, the moiré potential has little effect, and the signal difference is negligible. Notably, this signal difference persists and remains sizable even under realistic line broadening δ, which is a key characteristic of moiré polaritons. These numerical results agree qualitatively with previous SNOM experiments.^41^ The spatial variation of near-field response remains robust against line width broadening, ensuring reliable experimental detections.

We also calculate the PhP spectrum of 3.89° twisted bilayer MoTe_2_ (SI Section 3.4), which has aroused great interest recently.^27,28^ Compared with hBN, the gaps between mini-branches in MoTe_2_ are smaller, and its critical frequency ω_0_/(2π) ≈ 7.2 THz is also lower, due to the heavier atomic mass. However, some basic properties are qualitatively the same. For example, the spectrum also consists of a linearly dispersive dominant branch and some flat mini-branches, and the intensities become weaker as ω deviates from ω_0_ to lower frequencies.

Finally, we introduce a continuum model that can reproduce the same physics. This model generalizes Huang’s continuum eqs 1 and 2 and is more computationally efficient than the lattice model since it contains only a few parameters (SI Sections 4.2, 4.3). In this model, the vibration field consists of layer- and (commensurate wavevector) ***Q***-resolved terms: ***W*** = *∑*_***Q*** *l*_ ***W***_***Q*** *l*_ *e* ^*i* ***Q*** · ***r***^. Each ***W***_***Q*** *l*_ has a unique resonance frequency ω_***Q*** *l*_. These components couple to each other and to the electric field: ${Ẅ}_{Ql}=-\underset{Q^{\prime }l^{\prime }}{\sum }D_{Ql,Q^{\prime }l^{\prime }}W_{Q^{\prime }l^{\prime }}+\gamma E_{Q}$ , and the polarization is ***P*** = *∑*_***Q*** *l*_γ ***W***_***Q*** *l*_ *e* ^*i* ***Q*** · ***r***^, where γ is the same as γ_12_ in eq 1. The matrix D takes nonzero elements only for $\left|Q-Q^{\prime }\right|\leq \frac{4\pi }{\sqrt{3}L_{\theta }}$ .^76^ The diagonal terms $D_{Ql,Ql}$ are $\omega_{Ql}^{2}$ , and the off-diagonal terms $D_{Ql,Q^{\prime }l^{\prime }}$ hybridize different components ***W***_***Q*** *l*_. To understand why it works, we note that the continuum model essentially describes a system of coupled harmonic oscillators driven by an external field (SI Section 4.1). The elastic coupling (moiré potential) turns the single-pole susceptibility (eq 2) into the multipole one (eq 6).^33^ This means that the long-wavelength optical components are scattered and redistributed among the phonon branches that are backfolded to the mBZ center. In general, the model parameters depend on lattice relaxations in larger supercells, which will be systematically studied in the future.

Following the spirit of Huang’s theory, we have derived a set of macroscopic equations to understand 2D PhPs. For moiré systems, the eigenequation couples different momenta together, resulting in multiple branches of inhomogeneous PhP modes with moiré patterns. The theoretical proposal was numerically verified using the lattice model. Many PhP bands are obtained, each carrying a unique EM wave that differs in polarization and localization. The inhomogeneous multibranch physics can be understood by generalizing Huang’s theory to that of coupled harmonic oscillators. In this study, we have calculated only for two specific moiré systems with relatively small supercells. There remains plenty of room to explore the dependence of optical properties on the material parameters. For example, samples with supercells comparable to achievable light wavelengths are more promising for experiments.^41^ The properties of moiré PhPs could be engineered via the twisting angle, which would conceivably balance the moiré potential strength against the separation of folded phonon bands—a systematic study of this dependence is an important direction for future studies. The spatial localization of EM waves and the tunability of their wavelengths and frequencies represent fascinating features of 2D optics. If such modes can be excited efficiently, they could provide flexible driven potentials that differ completely from traditional light fields.^70,77^ We defer these explorations to future studies.

## Supplementary Material



## Notes

(I) The derivation of the macroscopic 2D PhP theory; (II) the lattice model of 2D PhP; (III) the lattice model of moiré PhP; (IV) the macroscopic theory of moiré PhPs; (V) more details about the moiré response function; (VI) the interatomic force constants used in MD simulations (PDF)
