# Supplementary material for: Two-Dimensional Moiré Phonon Polaritons
Source: Nano Lett. 2025 Oct 16;25(43):15460–7. doi: 10.1021/acs.nanolett.5c03046 (PMC12576835; doi:10.1021/acs.nanolett.5c03046)
Supplement: Supplementary file 1 [file nl5c03046_si_001.pdf]

Supporting Information for "Two-dimensional moiré phonon polaritons"

Hao Shi[1,2], Chu Li[2], Ding Pan[2,3], and Xi Dai[1,2]

[1]New Cornerstone Science Laboratory, Department of Physics, The Hong Kong University of Science and Technology, Hong Kong, China
[1]Department of Physics, The Hong Kong University of Science and Technology, Clear Water Bay, Hong Kong, China
[2]Department of Chemistry, The Hong Kong University of Science and Technology, Clear Water Bay, Hong Kong, China

Contents

1 Macroscopic theory of 2D PhP **2**
1.1 Huang's equation in 2D 2
1.2 Non-retarded solutions 3
1.3 Guided modes: 2D EM 3
1.4 Radiative solutions: 3D EM 4
1.5 Revisit 2D PhP as a light reflection and refraction problem 4

2 Lattice model of 2D PhP in simple polar systems **5**
2.1 Lattice dynamics of 2D polar systems 5
2.2 Relation to the macroscopic theory 7
2.3 Force constant of monolayer hBN 9

3 Lattice model of 2D PhP in moiré polar systems **9**
3.1 Equation of motion and polarization in moiré superlattice 9
3.2 Moiré polaritons 12
3.3 Moiré PhP dispersion against phonon linewidth 13
3.4 PhP spectrum of twisted bilayer MoTe2 system 14

4 Macroscopic theory of moiré PhP **15**
4.1 A toy model: coupled harmonic oscillators 15
4.2 The continuum model for moiré phonon 16
4.3 The continuum model for moiré PhP 19

5 More details about the moiré response function **20**
5.1 Derivation in quantum case 20
5.2 Non-locality and inhomogeneity 22
5.3 Symmetry properties 22
5.4 Representation in moiré-less basis 23

6 Interatomic force constants **24**

1 Macroscopic theory of 2D PhP

1.1 Huang's equation in 2D

Consider a 2D polar sheet placed at $z = 0$ of a dielectric with permittivity $\varepsilon_0\varepsilon$ (in this document we generalize the vacuum case) and permeability $\mu = 1$ [Fig. 1(a) of the main text]. We import a continuum vibrational field $\boldsymbol{W}$ defined in the plane. For diatomic ionic crystals like hBN it is connected to the ion displacements through $\boldsymbol{W} \propto \boldsymbol{u}_+ - \boldsymbol{u}_-$. Under an electric field $\boldsymbol{E}$, $\boldsymbol{W}$ satisfies the equation of motion of a driven oscillator (neglect dissipation)

$$\ddot{\boldsymbol{W}} = -\omega_0^2 \boldsymbol{W} + \gamma_{12}\boldsymbol{E}_t, \tag{1}$$

where ω_0 is the optical phonon frequency resulting from elastic forces, $\boldsymbol{E}_t$ is the in-plane part of $\boldsymbol{E}$ at $z = 0$. The surface polarization density (in-plane dipole moment per unit area) is denoted by $\boldsymbol{P}$. In 2D it originates mainly from the relative ionic displacement (rigid-ion approximation)

$$\boldsymbol{P} = \gamma_{21}\boldsymbol{W}. \tag{2}$$

The parameters $\gamma_{12} = \gamma_{21}$ due to Onsager relations (or can be inferred from microscopic models). For clarity we set $\gamma_{12}^2 = \gamma_{21}^2 = \epsilon_0 T$. The above two equations are 2D version of Huang's equations. Their microscopic origin is derived in Sec. 2.2. They should be solved together with Maxwell's equations. These equations govern the form of EM waves in the dielectric $z \neq 0$, and, at the interface $z = 0$ reduce to boundary conditions (BC):

$$\text{1st}: \quad \boldsymbol{E}_t^+ - \boldsymbol{E}_t^- = \boldsymbol{0}, \tag{3a}$$

$$\text{2nd}: \quad \varepsilon_0\varepsilon(E_z^+ - E_z^-) = \rho, \tag{3b}$$

$$\text{3rd}: \quad \boldsymbol{B}_t^+ - \boldsymbol{B}_t^- = \mu_0 \boldsymbol{J} \times \boldsymbol{e}_z, \tag{3c}$$

$$\text{4th}: \quad B_z^+ - B_z^- = 0, \tag{3d}$$

where ρ and $\boldsymbol{J}$ are surface charge and current densities, and '$\pm$' indicates fields just above or below the sheets, e.g., $\boldsymbol{E}^\pm = \boldsymbol{E}(z = 0^\pm)$. We study solutions of the form

$$\boldsymbol{E}, \boldsymbol{W} \propto e^{i\boldsymbol{q}\cdot\boldsymbol{r} - i\omega t}, \tag{4}$$

where $\boldsymbol{r} = (x, y)$ and $\boldsymbol{q}$ are *in-plane* position and momentum, respectively. The in-plane dipole current and charge density

$$\boldsymbol{J} = \partial\boldsymbol{P}/\partial t = -i\omega\boldsymbol{P}, \quad \rho = -i\boldsymbol{q}\cdot\boldsymbol{P}. \tag{5}$$

Then the 2nd and 3rd BCs become

$$\varepsilon_0\varepsilon(E_z^+ - E_z^-) = -i\boldsymbol{q}\cdot\boldsymbol{P}, \tag{6}$$

$$\partial_z\boldsymbol{E}_t^+ - \partial_z\boldsymbol{E}_t^- - i\boldsymbol{q}(E_z^+ - E_z^-) = -\mu_0\omega^2\boldsymbol{P}, \tag{7}$$

while the 4th BC coincides with the 1st one. In the dielectric ($z \neq 0$), the $\boldsymbol{E}$ field obeys the divergence law and the wave equation

$$\nabla\cdot\boldsymbol{E} = i\boldsymbol{q}\cdot\boldsymbol{E}_t + \partial_z E_z = 0, \tag{8}$$

$$(\nabla^2 - \varepsilon\partial_t^2)\boldsymbol{E} = \left(\partial_z^2 - q^2 + \varepsilon\frac{\omega^2}{c^2}\right)\boldsymbol{E} = \boldsymbol{0}. \tag{9}$$

Depending on the sign of

$$\lambda^2 = q^2 - \varepsilon\frac{\omega^2}{c^2}, \tag{10}$$

the solutions can be divided into guided or radiative modes. For $\omega \neq \omega_0$, the susceptibility can be defined

$$\boldsymbol{P} = \varepsilon_0\Pi(\omega)\boldsymbol{E}_t, \quad \Pi(\omega) = \frac{T}{\omega_0^2 - \omega^2}. \tag{11}$$

1.2 Non-retarded solutions

Let us first consider the non-retarded (static) limit without *external* field. In this limit, c is taken to infinity, so $\lambda = q$. Furthermore, we only need to consider the 1st and 2nd BCs. At $q = 0$, pure oscillation $\boldsymbol{W} \neq \boldsymbol{0}$ happens and $\boldsymbol{E} = \boldsymbol{0}$, with the transverse and longitudinal oscillations sharing the same frequency ω_0. For $q > 0$, the transverse pure oscillation with frequency $\omega_{\mathrm{TO}} = \omega_0$ is still a solution (TO mode), but the longitudinal mode will move with built-in electric field. For the longitudinal mode, $\boldsymbol{E}_t$ has nonzero longitudinal component $E_\parallel$. Suppose $E_\parallel \propto e^{i\boldsymbol{q}\cdot\boldsymbol{r}-\lambda|z|-i\omega t}$. In the static limit, Eq. (8) gives $E_z^\pm = \pm iE_\parallel$, and the 2nd BC gives $2iE_\parallel = -iqP_\parallel/(\epsilon_0\varepsilon)$. Combining this with Eq. (11) yields the static longitudinal mode condition:

$$1 + \frac{q}{2\varepsilon}\Pi(\omega) = 0, \tag{12}$$

from which we get the longitudinal dispersion

$$\omega_{\mathrm{LO}} = \omega_0\sqrt{1 + \frac{qT}{2\varepsilon\omega_0^2}} \approx \omega_0 + \frac{qT}{4\varepsilon\omega_0}. \tag{13}$$

The static transverse and longitudinal phonon dispersions are shown in Fig. 1(b) of the main text using dashed lines. We see that the above model, although simple, captures both the degeneracy of TO and LO phonons at Γ point and the linear LO-TO splitting in the long-wavelength regime, which are typical properties of 2D polar systems [1, 2, 3]. We notice that only in the non-retarded limit, the condition (12) for LO phonon coincides with that for the 2D TM polariton studied before [2, 4].

1.3 Guided modes: 2D EM

First let us focus on the regime $\lambda^2 > 0$. We focus on the case $\omega \neq \omega_0$, where Eq. (11) can be used to eliminate $\boldsymbol{W}$ ($\omega = \omega_0$ leads to the trivial solution $\boldsymbol{W} = \boldsymbol{E} = \boldsymbol{0}$). Setting $\lambda = \sqrt{q^2 - \varepsilon\omega^2/c^2} > 0$, we may assume the following localized $\boldsymbol{E}$ field

$$\boldsymbol{E}(\boldsymbol{r}, z, t) = \begin{cases} (\boldsymbol{E}_t + E_z^+\boldsymbol{e}_z)e^{i\boldsymbol{q}\cdot\boldsymbol{r}-\lambda z-i\omega t} & z > 0 \\ (\boldsymbol{E}_t + E_z^-\boldsymbol{e}_z)e^{i\boldsymbol{q}\cdot\boldsymbol{r}+\lambda z-i\omega t} & z < 0 \end{cases}, \tag{14}$$

where $\boldsymbol{E}_t = E_\parallel\boldsymbol{e}_{\boldsymbol{q}} + E_\perp\boldsymbol{e}_z \times \boldsymbol{e}_{\boldsymbol{q}}$. Here, $\boldsymbol{e}_{\boldsymbol{q}}$ is the unit vector along $\boldsymbol{q}$. The divergence theorem gives

$$E_z^+ = -E_z^- = iqE_\parallel/\lambda. \tag{15}$$

The 2nd BC (6) becomes

$$2\frac{iqE_\parallel}{\lambda} = -\frac{iqP_\parallel}{\epsilon_0\epsilon} \Rightarrow \left[1 + \frac{\lambda}{2\varepsilon}\Pi(\omega)\right]E_\parallel = 0, \tag{16}$$

The longitudinal part of the 3rd BC (7) is equivalent to the 2nd one, while the transverse part reads

$$2\lambda qE_\perp = \mu_0\omega^2 qP_\perp \Rightarrow \left[1 - \frac{1}{2\lambda}\frac{\omega^2}{c^2}\Pi(\omega)\right]E_\perp = 0. \tag{17}$$

We see that in this isotropic toy model, the solutions for $E_\parallel$ and $E_\perp$ are decoupled. In realistic models, especially in moiré materials, they are in general coupled.

Consider first the case $E_\parallel \neq 0$, $E_\perp = 0$. This gives a $\boldsymbol{B}$ field polarized in the direction perpendicular to $\boldsymbol{e}_{\boldsymbol{q}}$,

$$\begin{aligned} \boldsymbol{E}^\pm &= \left(E_\parallel\boldsymbol{e}_{\boldsymbol{q}} \pm i\frac{q}{\lambda}E_\parallel\boldsymbol{e}_z\right)e^{i\boldsymbol{q}\cdot\boldsymbol{r}-i\omega t-\lambda|z|}, \\ \boldsymbol{B}^\pm &= \mp i\frac{\omega\varepsilon}{\lambda c^2}E_\parallel\boldsymbol{e}_z \times \boldsymbol{e}_{\boldsymbol{q}}e^{i\boldsymbol{q}\cdot\boldsymbol{r}-i\omega t-\lambda|z|}, \end{aligned} \tag{18}$$

which is the transverse magnetic (TM) mode. In this case this eigen equation reduces to the TM mode condition

$$\mathrm{TM}: \quad 1 + \frac{\lambda}{2\varepsilon}\Pi(\omega) = 0. \tag{19}$$

Since $\lambda > 0$, it has a solution when $\Pi(\omega) < 0$, i.e., when $\omega > \omega_0$. The dispersion reads

$$q = \sqrt{\varepsilon \frac{\omega^2}{c^2} + \varepsilon^2 \frac{(\omega^2 - \omega_0^2)^2}{T^2/4}}. \tag{20}$$

At $\omega = \omega_0$, $q = q_0 = \sqrt{\varepsilon}\omega_0/c$, the group velocity $v_0 = \mathrm{d}\omega/\mathrm{d}q|_{q=q_0} = c/\sqrt{\varepsilon}$, i.e., the TM mode is tangential to the light cone. As shown in Fig. 1(b), when $q \gg q_0$, the dispersion becomes linear and asymptotically approaches the static LO mode Eq. (13).

Then consider the case $E_\parallel = 0$, $E_\perp \neq 0$, which corresponds to the transverse electric (TE) mode

$$\begin{aligned} \boldsymbol{E}^\pm &= E_\perp \boldsymbol{e}_z \times \boldsymbol{e}_{\boldsymbol{q}} e^{i\boldsymbol{q}\cdot\boldsymbol{r} - i\omega t - \lambda|z|}, \\ \boldsymbol{B}^\pm &= \left(\pm \frac{\lambda}{i\omega} E_\perp \boldsymbol{e}_{\boldsymbol{q}} + \frac{q}{\omega} E_\perp \boldsymbol{e}_z \right) e^{i\boldsymbol{q}\cdot\boldsymbol{r} - i\omega t - \lambda|z|}. \end{aligned} \tag{21}$$

The dispersion obeys the TE mode condition

$$\mathrm{TE}: \quad 1 - \frac{1}{2\lambda} \frac{\omega^2}{c^2} \Pi(\omega) = 0, \tag{22}$$

which has solutions when $\Pi(\omega) > 0$, i.e., when $\omega < \omega_0$. The dispersion reads

$$q = \frac{\omega}{c} \sqrt{\varepsilon + \frac{\omega^2}{c^2} \frac{T^2/4}{(\omega_0^2 - \omega^2)^2}}. \tag{23}$$

When $q < q_0$, the TE mode resembles light: the dispersion closely follows the light cone, hence with a tiny λ and weak localization. When $q > q_0$, it turns almost into pure lattice oscillations: the dispersion remains very close to the static TO line $\omega = \omega_0$ and the EM fields are weak and extremely localized at the surface.

The above TM and TE modes are typical 2D EM waves with their energy constrained along the z-axis. Such modes, accompanied by polarizable collective modes, exist ubiquitously in 2D materials or at the interfaces of 3D materials. The eigenmode conditions (19) and (22) are general. For example, when discussing 2D plasmon polaritons one only needs to replace $\Pi(\omega)$ by its plasmon version. It is also true that the sign of $\Pi(\omega)$ governs whether the mode is TM or TE.

1.4 Radiative solutions: 3D EM

Then we consider the case $\lambda^2 < 0$, i.e., the left side of the light cone. This means the wave vector along the z-axis is real, and we take $\lambda = -i\sqrt{\varepsilon\omega^2/c^2 - q^2} = -ik_z$. In other words, EM fields occupy the whole 3D space without decaying. In this case, we can no longer expect unidirectional waves, like those in Eq. (14), to exist in both $z > 0$ and $z < 0$ regions. Otherwise, if $\boldsymbol{E}^\pm = (\boldsymbol{E}_t + E_z^\pm \boldsymbol{e}_z) e^{i\boldsymbol{q}\cdot\boldsymbol{r} \pm ik_z z - i\omega t}$, following the derivation for $\lambda^2 > 0$ case, the resulting eigen equations are the same as Eqs. (19) and (22), which have no solution since λ is imaginary. Alternatively, if we take $\boldsymbol{E}^\pm = (\boldsymbol{E}_t + E_z^\pm \boldsymbol{e}_z) e^{i\boldsymbol{q}\cdot\boldsymbol{r} + ik_z z - i\omega t}$, we find that $E_z^+ = E_z^-$. The 3rd BC gives $\boldsymbol{P} = \boldsymbol{0}$ and thus $\boldsymbol{W} = \boldsymbol{E} = \boldsymbol{0}$, which is also trivial. Instead, the $\boldsymbol{E}$ field must be composed of waves propagating in multiple directions in at least one half-space. A typical solution is the light incidence setup, where the 2D sheet is treated as a scattering potential. In the half-space containing the light source, plane waves propagate in two directions (incident and reflected). For this setup, a non-trivial solution exists for all $(\boldsymbol{q}, \omega)$ except when $\omega = \omega_0$, corresponding to the continuous spectrum in the dispersion plots. We will solve these in the next subsection, where the light incidence setup is generalized to contain both guided and radiative modes.

1.5 Revisit 2D PhP as a light reflection and refraction problem

Both the localized and radiative modes can be understood from the light reflection and refraction viewpoint. This approach is directly connected to experimental techniques for exciting these modes and offers greater numerical convenience for revealing their dispersions. We denote the incident, reflected, and refracted light as $\boldsymbol{E}^i$, $\boldsymbol{E}^r$, and $\boldsymbol{E}^t$. Then we assume the light is incident from $z < 0$,

$$\begin{aligned} \boldsymbol{E}^i &= (\boldsymbol{E}_t^i + iqE_\parallel^i/\lambda \boldsymbol{e}_z) e^{i\boldsymbol{q}\cdot\boldsymbol{r} - \lambda z - i\omega t}, \\ \boldsymbol{E}^r &= (\boldsymbol{E}_t^r - iqE_\parallel^r/\lambda \boldsymbol{e}_z) e^{i\boldsymbol{q}\cdot\boldsymbol{r} + \lambda z - i\omega t}, \\ \boldsymbol{E}^t &= (\boldsymbol{E}_t^t + iqE_\parallel^t/\lambda \boldsymbol{e}_z) e^{i\boldsymbol{q}\cdot\boldsymbol{r} - \lambda z - i\omega t}, \end{aligned} \tag{24}$$

where the in-plane part $\boldsymbol{E}_t^l = E_{\|}^l \boldsymbol{e_q} + E_{\perp}^l \boldsymbol{e}_z \times \boldsymbol{e_q}$, $l = i, r, t$. Here λ is allowed to take real or imaginary values,

$$\lambda = \begin{cases} -i\sqrt{\varepsilon\frac{\omega^2}{c^2} - q^2}, & q < \sqrt{\varepsilon}\omega/c \\ \sqrt{q^2 - \varepsilon\frac{\omega^2}{c^2}}, & q > \sqrt{\varepsilon}\omega/c \end{cases}, \tag{25}$$

depending on whether the EM wave is radiative or guided. Note that for the guided case ($q > \sqrt{\varepsilon}\omega/c$), an incident wave from a prism is also evanescent (attenuated). Such a wave can be generated using the Otto configuration [5]. Then the 1st BC reads

$$\boldsymbol{E}_t^t = \boldsymbol{E}_t^i + \boldsymbol{E}_t^r. \tag{26}$$

The 2nd BC reads

$$\boldsymbol{e_q} \cdot \left[\boldsymbol{E}_t^t - \boldsymbol{E}_t^i + \boldsymbol{E}_t^r + \frac{\lambda}{\varepsilon}\Pi(\omega)\boldsymbol{E}_t^t\right] = 0. \tag{27}$$

The longitudinal part of the 3rd BC is still equivalent to the above one, and the transverse part reads

$$\boldsymbol{e_q} \times \left[\boldsymbol{E}_t^t - \boldsymbol{E}_t^i + \boldsymbol{E}_t^r - \frac{1}{\lambda}\frac{\omega^2}{c^2}\Pi(\omega)\boldsymbol{E}_t^t\right] = 0. \tag{28}$$

From the above equations, we can express $\boldsymbol{E}^t$ and $\boldsymbol{E}^r$ using the incident light $\boldsymbol{E}^i$,

$$\boldsymbol{E}_t^t = T(\boldsymbol{q}, \omega)\boldsymbol{E}_t^i, \quad \boldsymbol{E}_t^r = R(\boldsymbol{q}, \omega)\boldsymbol{E}_t^i, \tag{29}$$

and the transmission and reflection matrices are (written in the basis of $E_{\|}^l$, $E_{\perp}^l$, where I is the identity matrix)

$$T(\boldsymbol{q}, \omega) = \begin{pmatrix} 1 + \frac{\lambda}{2\varepsilon}\Pi(\omega) & \\ & 1 - \frac{1}{2\lambda}\frac{\omega^2}{c^2}\Pi(\omega) \end{pmatrix}^{-1}, \tag{30a}$$

$$R(\boldsymbol{q}, \omega) = T(\boldsymbol{q}, \omega) - I. \tag{30b}$$

When $(\boldsymbol{q}, \omega)$ lies in the radiative regime, the above equations describe the usual transmission and reflection problem discussed in Sec. 1.4. When $(\boldsymbol{q}, \omega)$ lies in the guided regime, the matrices T and R have poles corresponding exactly to the polariton dispersion discussed in Sec. 1.3. Poles indicate that E^t and E^r can be induced with an infinitesimal incidence E^i, indicating the excitation of intrinsic modes. We can visualize both the continuous spectrum and the discrete dispersion by plotting the transmission spectrum

$$\mathcal{L}(\boldsymbol{q}, \omega) = -\text{Im}[\det[T(\boldsymbol{q}, \omega + i\delta/2)]], \tag{31}$$

where the phonon linewidth δ is tiny and positive.

In this section we discuss only the moiré-less case. In moiré materials or heterostructures, multiple-pole response should be incorporated into Eqs. (1) and (2) to correctly describe the long-wavelength behavior. We leave such a generalization for Sec. 4.

2 Lattice model of 2D PhP in simple polar systems

2.1 Lattice dynamics of 2D polar systems

In this section we build the lattice theory for 2D polar systems. We focus on the moiré-less case here, but will generalize it to include moiré effects in the next section. In such a model, the short-ranged force among ions is described by the force constant $\Phi_{\alpha,\beta}$ introduced in Sec. 6, while the long-ranged Coulomb force (specifically, dipole-dipole interaction) is incorporated into the macroscopic electric fields. In the long-wavelength limit, it should reduce to the macroscopic Huang's theory introduced in Sec. 1.1.

Suppose we have a lattice where the ions' equilibrium positions are $\boldsymbol{r}_{i\alpha} = \boldsymbol{R}_i + \boldsymbol{\tau}_\alpha$, where $\boldsymbol{R}_i$ denotes the i-th unit cell, $\boldsymbol{\tau}_\alpha$ denotes the relative position of sublattice α. With the long-wavelength electric field

$\boldsymbol{E}(\boldsymbol{r}) = \boldsymbol{E}_{\boldsymbol{q}}(\omega)e^{i\boldsymbol{q}\cdot\boldsymbol{r}-i\omega t}$ ($\boldsymbol{q} \approx \boldsymbol{0}$), the equation of motion for lattice displacement $\boldsymbol{u}(\boldsymbol{r}_{i\alpha})$ is (we focus on in-plane dynamics in this study, so μ, ν take values in x, y only)

$$M_\alpha \ddot{u}_\mu(\boldsymbol{r}_{i\alpha}) + \sum_{j\beta\nu} \Phi_{\alpha\mu,\beta\nu}(\boldsymbol{r}_{i\alpha} - \boldsymbol{r}_{j\beta}) u_\nu(\boldsymbol{r}_{j\beta}) - Z_\alpha e E_{\boldsymbol{q},\mu}(\omega) e^{i\boldsymbol{q}\cdot\boldsymbol{r}_{i\alpha}-i\omega t} = 0, \tag{32}$$

where Z_α and M_α are effective (dimensionless) charge and mass (which are assumed isotropic) of sublattice α, e is the elementary charge, and $\Phi_{\alpha,\beta}$ is the force constant matrix for nearby ions. In the absence of $\boldsymbol{E}$, the above equation reduces to the harmonic equation of motion in the usual phonon problem. The polarization density is defined as [6, 7]

$$\boldsymbol{P}(\boldsymbol{r}) = \sum_{i\alpha} Z_\alpha e \boldsymbol{u}(\boldsymbol{r}_{i\alpha}) \delta(\boldsymbol{r} - \boldsymbol{r}_{i\alpha}), \tag{33}$$

where $\delta(\boldsymbol{r} - \boldsymbol{r}_0)$ is the 2D Dirac delta function. The above two equations are the lattice version of Eqs. (1), (2). The linearity of the oscillators allows for an analytical solution of $\boldsymbol{u}(\boldsymbol{r}_{i\alpha})$ in the presence of the $\boldsymbol{E}$ field. Suppose $\boldsymbol{u}$ can be expanded as

$$\boldsymbol{u}(\boldsymbol{r}_{i\alpha}) = \sum_a \frac{1}{\sqrt{M_\alpha}} e^{i\boldsymbol{q}\cdot\boldsymbol{r}_{i\alpha}-i\omega t} \boldsymbol{e}_{\alpha,a}(\boldsymbol{q}) B_a(\boldsymbol{q}, \omega), \tag{34}$$

where $\boldsymbol{e}_a(\boldsymbol{q})$ is the displacement vector of the a-th eigenmode with frequency $\omega_{\boldsymbol{q}a}$, satisfying

$$\sum_{\beta\nu} D_{\alpha\mu,\beta\nu}(\boldsymbol{q}) e_{\beta\nu,a}(\boldsymbol{q}) = \omega_{\boldsymbol{q}a}^2 e_{\alpha\mu,a}(\boldsymbol{q}), \tag{35}$$

and the orthogonality

$$\sum_{\alpha\mu} e^*_{\alpha\mu,a}(\boldsymbol{q}) e_{\alpha\mu,a'}(\boldsymbol{q}) = \delta_{aa'}, \quad \sum_a e^*_{\alpha\mu,a}(\boldsymbol{q}) e_{\beta\nu,a}(\boldsymbol{q}) = \delta_{\alpha\beta}\delta_{\mu\nu}, \tag{36}$$

where the k-space dynamical matrix reads

$$D_{\alpha,\beta}(\boldsymbol{q}) = \sum_j \frac{\Phi_{\alpha,\beta}(\boldsymbol{r}_{i\alpha} - \boldsymbol{r}_{j\beta})}{\sqrt{M_\alpha M_\beta}} e^{i\boldsymbol{q}\cdot(\boldsymbol{r}_{j\beta} - \boldsymbol{r}_{i\alpha})}. \tag{37}$$

The goal is to solve $B_a(\boldsymbol{q}, \omega)$. Plugging Eq. (34) into Eq. (32), we get

$$-\omega^2 \sum_a e_{\alpha\mu,a}(\boldsymbol{q}) B_a(\boldsymbol{q}, \omega) + \sum_{a\beta\nu} D_{\alpha\mu,\beta\nu}(\boldsymbol{q}) e_{\beta\nu,a}(\boldsymbol{q}) B_a(\boldsymbol{q}, \omega) - \frac{Z_\alpha e}{\sqrt{M_\alpha}} E_{\boldsymbol{q},\mu} = 0. \tag{38}$$

Then using Eqs. (35), (36), we get [recall that $\boldsymbol{E}_t$ is the in-plane part of $\boldsymbol{E}(z = 0)$]

$$(\omega_{\boldsymbol{q}a}^2 - \omega^2) B_a(\boldsymbol{q}, \omega) = e \boldsymbol{S}^*_a(\boldsymbol{q}) \cdot \boldsymbol{E}_{\boldsymbol{q},t}, \tag{39}$$

where we have defined the S matrix

$$\boldsymbol{S}_a(\boldsymbol{q}) = \sum_\alpha \frac{Z_\alpha \boldsymbol{e}_{\alpha,a}(\boldsymbol{q})}{\sqrt{M_\alpha}}. \tag{40}$$

When $\omega \neq \omega_0$ (which is guaranteed in numerics by introducing a tiny linewidth $\omega + i\delta/2$), we can solve for $B_a(\boldsymbol{q}, \omega)$ and plug it into Eq. (34) to obtain $\boldsymbol{u}$.

To apply macroscopic Maxwell's equations, we need to derive the continuous field $\boldsymbol{P}(\boldsymbol{r})$ from the lattice version in Eq. (33). This process is done by expanding Eq. (33) in a Fourier series $\boldsymbol{P}_{\boldsymbol{q}+\boldsymbol{b}}$ ($\boldsymbol{b}$ spans the reciprocal lattice) [6] and retaining only the leading term with $\boldsymbol{b} = \boldsymbol{0}$. Terms with $\boldsymbol{b} \neq \boldsymbol{0}$ are redundant because they detail the information within atomic unit cell and are thus not responsible for long-wavelength physics. So

$$\boldsymbol{P}(\boldsymbol{r}) = \sum_{\boldsymbol{b}} \boldsymbol{P}_{\boldsymbol{q}+\boldsymbol{b}}(\omega) e^{i(\boldsymbol{q}+\boldsymbol{b})\cdot\boldsymbol{r}-i\omega t} \to \boldsymbol{P}(\boldsymbol{r}) = \boldsymbol{P}_{\boldsymbol{q}}(\omega) e^{i\boldsymbol{q}\cdot\boldsymbol{r}-i\omega t}. \tag{41}$$

Then $\boldsymbol{P}_{\boldsymbol{q}}(\omega)$ can be calculated as

$$\boldsymbol{P}_{\boldsymbol{q}}(\omega) = \frac{1}{N_{\text{tot}}\Omega_0}\int d^2\boldsymbol{r}\boldsymbol{P}(\boldsymbol{r})e^{-i\boldsymbol{q}\cdot\boldsymbol{r}+i\omega t} = \frac{1}{N_{\text{tot}}\Omega_0}\sum_{i\alpha} Z_\alpha e\boldsymbol{u}(\boldsymbol{r}_{i\alpha})e^{-i\boldsymbol{q}\cdot\boldsymbol{r}_{i\alpha}+i\omega t} = \frac{e}{\Omega_0}\sum_a \boldsymbol{S}_a(\boldsymbol{q})B_a(\boldsymbol{q},\omega), \tag{42}$$

where Ω_0 is the area of the atomic unit cell and N_{tot} is the total number of unit cells. Using Eq. (39), we obtain

$$P_{\boldsymbol{q},\mu}(\omega) = \varepsilon_0\sum_\nu \Pi_{\mu\nu}(\boldsymbol{q},\omega)E_{\boldsymbol{q},\nu}(\omega), \tag{43}$$

where the in-plane susceptibility is a 2 by 2 matrix,

$$\Pi_{\mu\nu}(\boldsymbol{q},\omega) = \frac{e^2}{\varepsilon_0\Omega_0}\sum_a \frac{[\boldsymbol{S}_a(\boldsymbol{q})]_\mu[\boldsymbol{S}_a^*(\boldsymbol{q})]_\nu}{\omega_{\boldsymbol{q}a}^2-\omega^2}. \tag{44}$$

This result is also valid in the quantum case. By treating phonons as bosons, we will rederive this result for moiré systems using quantum linear response theory in Sec. 5.1. All the derivations of PhP conditions follow exactly the same procedure as in Secs. 1.3, 1.4, 1.5, since the macroscopic response function has already been obtained.

2.2 Relation to the macroscopic theory

Now it is time to relate the lattice model to the macroscopic model introduced in Sec. 1.1. We still focus on the moiré-less case. In the long-wavelength limit $|\boldsymbol{q}| \sim \omega_0/c$, the response function is almost isotropic and dispersionless, i.e.,

$$\Pi_{\mu\nu}(\boldsymbol{q},\omega) \approx \delta_{\mu\nu}\Pi(\omega). \tag{45}$$

The nonzero contribution comes from the LO and TO modes (for acoustic modes, the polarization from opposite ions cancels due to in-phase oscillation), which gives the isotropic response

$$\Pi(\omega) = \frac{e^2}{\varepsilon_0\Omega_0}\lim_{\boldsymbol{q}\to\boldsymbol{0}}\frac{|\boldsymbol{e}_{\boldsymbol{q}}\cdot\boldsymbol{S}_{\text{LO}}(\boldsymbol{q})|^2}{\omega_{\boldsymbol{q},\text{LO}}^2-\omega^2} = \frac{e^2}{\varepsilon_0\Omega_0}\lim_{\boldsymbol{q}\to\boldsymbol{0}}\frac{|(\boldsymbol{e}_z\times\boldsymbol{e}_{\boldsymbol{q}})\cdot\boldsymbol{S}_{\text{TO}}(\boldsymbol{q})|^2}{\omega_{\boldsymbol{q},\text{TO}}^2-\omega^2}. \tag{46}$$

Compared with Eq. (11), we recognize

$$\omega_0 = \omega_{\boldsymbol{0},\text{LO}} = \omega_{\boldsymbol{0},\text{TO}}, \tag{47a}$$

$$T = \frac{e^2}{\varepsilon_0\Omega_0}\lim_{\boldsymbol{q}\to\boldsymbol{0}}[\boldsymbol{e}_{\boldsymbol{q}}\cdot\boldsymbol{S}_{\text{LO}}(\boldsymbol{q})]^2 = \frac{e^2}{\varepsilon_0\Omega_0}\lim_{\boldsymbol{q}\to\boldsymbol{0}}[(\boldsymbol{e}_z\times\boldsymbol{e}_{\boldsymbol{q}})\cdot\boldsymbol{S}_{\text{TO}}(\boldsymbol{q})]^2. \tag{47b}$$

Substituting the above expressions into the static LO dispersion Eq. (13), we obtain the LO-TO splitting (the so-called non-analytical correction) in exact agreement with that in Ref. [1]:

$$\omega^2(\boldsymbol{q}) - \omega_0^2 = V(\boldsymbol{q})\frac{q^2}{\Omega_0}|\boldsymbol{e}_{\boldsymbol{q}}\cdot\boldsymbol{S}_{\text{LO}}(\boldsymbol{q})|^2_{\boldsymbol{q}\to\boldsymbol{0}}, \tag{48}$$

with the 2D Coulomb interaction $V(\boldsymbol{q}) = e^2/(2\varepsilon_0\varepsilon q)$. The screening term is missing here because we have used the zero-thickness approximation from the beginning [8]. From now on, for simplicity we focus on the monolayer hBN system. At $\boldsymbol{q}\approx\boldsymbol{0}$, for hBN the LO and TO phonons' eigenmode displacement vectors can be obtained using the invariance of the center of mass: $M_N\boldsymbol{u}_N + M_B\boldsymbol{u}_B \propto \sqrt{M_N}\boldsymbol{e}_N + \sqrt{M_B}\boldsymbol{e}_B = 0$, so

$$\begin{aligned}\boldsymbol{e}_{\text{LO}}(\boldsymbol{q}) &= [\boldsymbol{e}_{N,\text{LO}}(\boldsymbol{q}), \boldsymbol{e}_{B,\text{LO}}(\boldsymbol{q})] \approx \left(\sqrt{\frac{M_B}{M_N+M_B}}\boldsymbol{e}_{\boldsymbol{q}}, -\sqrt{\frac{M_N}{M_N+M_B}}\boldsymbol{e}_{\boldsymbol{q}}\right)^T,\\ \boldsymbol{e}_{\text{TO}}(\boldsymbol{q}) &= [\boldsymbol{e}_{N,\text{TO}}(\boldsymbol{q}), \boldsymbol{e}_{B,\text{TO}}(\boldsymbol{q})] \approx \left(\sqrt{\frac{M_B}{M_N+M_B}}\boldsymbol{e}_z\times\boldsymbol{e}_{\boldsymbol{q}}, -\sqrt{\frac{M_N}{M_N+M_B}}\boldsymbol{e}_z\times\boldsymbol{e}_{\boldsymbol{q}}\right)^T.\end{aligned} \tag{49}$$

At $\boldsymbol{q}=\boldsymbol{0}$, $\boldsymbol{e_q}=\boldsymbol{q}/|\boldsymbol{q}|$ is ill-defined, but we can fix $\boldsymbol{e_0}=\boldsymbol{e}_x$, $\boldsymbol{e}_z\times\boldsymbol{e_0}=\boldsymbol{e}_y$, i.e.,

$$\boldsymbol{e}_{\mathrm{LO}}(\boldsymbol{0})=\left(\sqrt{\frac{M_B}{M_N+M_B}}\boldsymbol{e}_x,-\sqrt{\frac{M_N}{M_N+M_B}}\boldsymbol{e}_x\right)^T,\boldsymbol{e}_{\mathrm{TO}}(\boldsymbol{0})=\left(\sqrt{\frac{M_B}{M_N+M_B}}\boldsymbol{e}_y,-\sqrt{\frac{M_N}{M_N+M_B}}\boldsymbol{e}_y\right)^T. \tag{50}$$

For monolayer hBN, the T parameter can then be analytically derived as

$$\lim_{\boldsymbol{q}\to\boldsymbol{0}}\boldsymbol{e_q}\cdot\boldsymbol{S}_{\mathrm{LO}}(\boldsymbol{q})=\lim_{\boldsymbol{q}\to\boldsymbol{0}}(\boldsymbol{e}_z\times\boldsymbol{e_q})\cdot\boldsymbol{S}_{\mathrm{TO}}(\boldsymbol{q})=\frac{Z_N}{\sqrt{M_N}}\sqrt{\frac{M_B}{M_N+M_B}}-\frac{Z_B}{\sqrt{M_B}}\sqrt{\frac{M_N}{M_N+M_B}}, \tag{51}$$

$$T=\frac{e^2}{\varepsilon_0\Omega_0}\frac{M_NM_B}{M_N+M_B}\left(\frac{Z_N}{M_N}-\frac{Z_B}{M_B}\right)^2=3.842\times10^{19}\ \mathrm{m/s^2}. \tag{52}$$

Using $\omega_0\approx2\pi\times49.4463$ THz, we get $T/(2\omega_0c)\approx2.06\times10^{-4}$, which is used to plot Fig. 1(b) in the main text. For the bilayer hBN without moiré potential (i.e., with no twist), T is doubled. Further, the macroscopic $\boldsymbol{W}$ field is related to the lattice dynamics through

$$\boldsymbol{W}(\boldsymbol{r},t)=\frac{1}{\sqrt{\Omega_0}}\sqrt{\frac{M_NM_B}{M_N+M_B}}[\boldsymbol{u}_N(\boldsymbol{r},t)-\boldsymbol{u}_B(\boldsymbol{r},t)]\propto\frac{1}{\sqrt{\Omega_0}}\sqrt{\frac{M_NM_B}{M_N+M_B}}\left[\frac{\boldsymbol{e}_N(\boldsymbol{q})}{\sqrt{M_N}}-\frac{\boldsymbol{e}_B(\boldsymbol{q})}{\sqrt{M_B}}\right]e^{i\boldsymbol{q}\cdot\boldsymbol{r}-i\omega t}, \tag{53}$$

where $\boldsymbol{u}_\alpha(\boldsymbol{r},t)$ is the continuum version [similar to Eq. (41)] of the displacement field for sublattice α.

We now derive the continuum Huang's equations Eqs. (1), (2). The key point is that, under the electric field, the optically active displacement field can be expanded using the field-free iLO/iTO modes [Eq. (34)], i.e.,

$$\boldsymbol{u}(\boldsymbol{r}_{i\alpha})=\frac{e^{i\boldsymbol{q}\cdot\boldsymbol{r}_{i\alpha}}}{\sqrt{M_\alpha}}(\boldsymbol{e}_{\alpha,\mathrm{LO}}(\boldsymbol{q}),\boldsymbol{e}_{\alpha,\mathrm{TO}}(\boldsymbol{q}))\begin{pmatrix}A_{\mathrm{LO}}\\A_{\mathrm{TO}}\end{pmatrix}. \tag{54}$$

Notice that we have absorbed the time dependence into $A_{\mathrm{LO/TO}}$. Then from Eq. (38) we know

$$\begin{aligned}\frac{Z_\alpha e}{M_\alpha}\boldsymbol{E_q}e^{i\boldsymbol{q}\cdot\boldsymbol{r}_{i\alpha}-i\omega t}&=\sum_a\frac{e^{i\boldsymbol{q}\cdot\boldsymbol{r}_{i\alpha}}}{\sqrt{M_\alpha}}\boldsymbol{e}_{\alpha,a}(\boldsymbol{q})\ddot{A}_a+\sum_a\omega_{\boldsymbol{q}a}^2\frac{e^{i\boldsymbol{q}\cdot\boldsymbol{r}_{i\alpha}}}{\sqrt{M_\alpha}}\boldsymbol{e}_{\alpha,a}(\boldsymbol{q})A_a\\&\approx\sum_a\frac{e^{i\boldsymbol{q}\cdot\boldsymbol{r}_{i\alpha}}}{\sqrt{M_\alpha}}\boldsymbol{e}_{\alpha,a}(\boldsymbol{q})\ddot{A}_a+\omega_0^2\sum_a\frac{e^{i\boldsymbol{q}\cdot\boldsymbol{r}_{i\alpha}}}{\sqrt{M_\alpha}}\boldsymbol{e}_{\alpha,a}(\boldsymbol{q})A_a\\&=\ddot{\boldsymbol{u}}(\boldsymbol{r}_{i\alpha})+\omega_0^2\boldsymbol{u}(\boldsymbol{r}_{i\alpha}),\end{aligned} \tag{55}$$

where we have adopted the Einstein approximation: $\omega_{\boldsymbol{q}a}=\omega_0$ (ω_0 is the degenerate eigen frequency of iLO/iTO modes at $\boldsymbol{q}=\boldsymbol{0}$) in the second line. The continuum version of the above equation of motion is simply $\ddot{\boldsymbol{u}}_\alpha(\boldsymbol{r},t)=-\omega_0^2\boldsymbol{u}_\alpha(\boldsymbol{r},t)+(Z_\alpha e/M_\alpha)\boldsymbol{E}_t(\boldsymbol{r},t)$. Then using the relation Eq. (53) we get Eq. (1)

$$\ddot{\boldsymbol{W}}(\boldsymbol{r},t)=-\omega_0^2\boldsymbol{W}(\boldsymbol{r},t)+\frac{e}{\sqrt{\Omega_0}}\sqrt{\frac{M_NM_B}{M_N+M_B}}\left(\frac{Z_N}{M_N}-\frac{Z_B}{M_B}\right)\boldsymbol{E}_t(\boldsymbol{r},t). \tag{56}$$

The relationship Eq. (2) is easily obtained through the continuum version of Eq. (33)

$$\boldsymbol{P}(\boldsymbol{r},t)=\frac{1}{\Omega_0}\sum_\alpha Z_\alpha e\boldsymbol{u}_\alpha(\boldsymbol{r},t)=\frac{Z_Ne}{\Omega_0}(\boldsymbol{u}_N-\boldsymbol{u}_B)=\frac{Z_Ne}{\sqrt{\Omega_0}}\sqrt{\frac{1}{M_N}+\frac{1}{M_B}}\boldsymbol{W}(\boldsymbol{r},t). \tag{57}$$

So we see the microscopic expressions for γ_{12}, γ_{21} appearing in Eqs. (1), (2) are

$$\gamma_{12}=\frac{e}{\sqrt{\Omega_0}}\sqrt{\frac{M_NM_B}{M_N+M_B}}\left(\frac{Z_N}{M_N}-\frac{Z_B}{M_B}\right)=\frac{Z_Ne}{\sqrt{\Omega_0}}\sqrt{\frac{1}{M_N}+\frac{1}{M_B}}=\gamma_{21}, \tag{58}$$

and $\gamma_{12}^2=\gamma_{21}^2=\varepsilon_0T$ is recovered. We notice that in deriving the continuum theory, the $\boldsymbol{q}$-dependence in the phonon level has been abandoned, which is an excellent approximation since the optical region $q\sim q_0=\omega_0/c$ is four to five orders smaller than the Brillouin zone size $1/a_0$. In other words, the $\boldsymbol{q}$-dispersion of PhP comes almost completely from light. We also note that for the moiré case, when Eq. (65) is satisfied, we can still abandon the $\bar{\boldsymbol{q}}$-dependence. However, it is necessary to retain the $\boldsymbol{Q}$-dependence of $\omega_{\bar{\boldsymbol{q}}+\boldsymbol{Q},b}$ (and dynamical matrix): $\omega_{\bar{\boldsymbol{q}}+\boldsymbol{Q},b}\approx\omega_{\boldsymbol{Q},b}$. It is such splitting of $\omega_{\boldsymbol{Q},b}$ that gives rise to the multiple branches of moiré PhP.

2.3 Force constant of monolayer hBN

Here we detail the lattice model used for monolayer hBN. The short-ranged elastic force constants (FC) $\Phi_{\alpha,\beta}(\boldsymbol{r}_{i\alpha}-\boldsymbol{r}_{j\beta})$ are used to generate the bare phonon dispersion and eigenmode displacement vectors. Moreover, this monolayer model provides the moiré-less basis in the continuum model we will derive in Secs. 4.2 and 4.3.

The monolayer hBN is a hexagonal lattice with lattice constant $a_0 = 2.504$ Å. The Bravais lattice vectors are $\boldsymbol{a}_1 = a_0(1/2, \sqrt{3}/2)$, $\boldsymbol{a}_2 = a_0(-1/2, \sqrt{3}/2)$. The nitride ($N$) and boron ($B$) atoms are located at $\boldsymbol{\tau}_N = -\boldsymbol{\tau}_B = (\boldsymbol{a}_1 + \boldsymbol{a}_2)/3 = (a_0/\sqrt{3})\boldsymbol{e}_y$ near the origin. The lattice has the C_{3z}, C_{2y}, and M_z symmetries. Notice that M_z results in the decoupling of in-plane and out-of-plane phonons of hBN monolayer, so we can consider only the in-plane parts. For simplicity we retain only the onsite, nearest-neighboring (n.n.), and next-nearest neighboring (n.n.n.) FCs. Each N (B) atom has 3 n.n. B (N) atoms, denoted by 3 relative vectors $(\boldsymbol{r}_{iN} - \boldsymbol{r}_{jB})_{\text{n.n.}} \in \{\boldsymbol{\delta}_1^0, \boldsymbol{\delta}_2^0, \boldsymbol{\delta}_3^0\}$, where $\boldsymbol{\delta}_1^0 = -(a_0/\sqrt{3})\boldsymbol{e}_y$; $\boldsymbol{\delta}_2^0 = C_{3z}\boldsymbol{\delta}_1^0$; $\boldsymbol{\delta}_3^0 = C_{3z}^2\boldsymbol{\delta}_1^0$. The n.n. FCs are (in x, y basis)

$$\Phi_{N,B}(\boldsymbol{\delta}_1^0) = \begin{pmatrix} t_{xx}^0 & \\ & t_{yy}^0 \end{pmatrix}, \quad \Phi_{N,B}(\boldsymbol{\delta}_2^0) = C_{3z}\Phi_{N,B}(\boldsymbol{\delta}_1^0)C_{3z}^{-1}, \quad \Phi_{N,B}(\boldsymbol{\delta}_3^0) = C_{3z}^{-1}\Phi_{N,B}(\boldsymbol{\delta}_1^0)C_{3z}. \tag{59}$$

The other n.n. FCs are obtained through $\Phi_{B,N}(-\boldsymbol{\delta}_j^0) = \Phi_{N,B}^T(\boldsymbol{\delta}_j^0)$ for $j = 1, 2, 3$ (T is the transpose). Each N (B) atom has 6 n.n.n. N (B) atoms, denoted by 6 relative vectors $(\boldsymbol{r}_{iN(B)} - \boldsymbol{r}_{jN(B)})_{\text{n.n.n.}} \in \{\boldsymbol{\delta}_j^1 (j = 1, 2, ..., 6)\}$, where $\boldsymbol{\delta}_1^1 = a_0\boldsymbol{e}_x$; $\boldsymbol{\delta}_j^1 = C_{6z}^{j-1}\boldsymbol{\delta}_1^1$. The n.n.n. FCs are

$$\begin{aligned} &\Phi_{N,N}(\boldsymbol{\delta}_1^1) = \begin{pmatrix} t_{xx}^1 & t_{xy}^1 \\ -t_{xy}^1 & t_{yy}^1 \end{pmatrix}, \quad \Phi_{N,N}(\boldsymbol{\delta}_3^1) = C_{3z}\Phi_{N,N}(\boldsymbol{\delta}_1^1)C_{3z}^{-1}, \quad \Phi_{N,N}(\boldsymbol{\delta}_6^1) = C_{3z}^{-1}\Phi_{N,N}(\boldsymbol{\delta}_1^1)C_{3z}, \\ &\Phi_{N,N}(\boldsymbol{\delta}_2^1) = \Phi_{N,N}^T(\boldsymbol{\delta}_5^1), \quad \Phi_{N,N}(\boldsymbol{\delta}_4^1) = \Phi_{N,N}^T(\boldsymbol{\delta}_1^1), \quad \Phi_{N,N}(\boldsymbol{\delta}_6^1) = \Phi_{N,N}^T(\boldsymbol{\delta}_3^1). \end{aligned} \tag{60}$$

The FCs among B atoms are $\Phi_{B,B}(\boldsymbol{\delta}_j^1) = \Phi_{N,N}^T(\boldsymbol{\delta}_j^1)$. Notice that $\Phi_{B,B}$ and $\Phi_{N,N}$ are actually independent, i.e., they are not related by any symmetry. In our present simple model, they are set to be related in this way, which is also supported by MD simulations. The onsite FCs are obtained through the sum rule

$$\Phi_{N,N}(\boldsymbol{0}) = -\sum_{j=1}^{3}\Phi_{N,B}(\boldsymbol{\delta}_j^0) - \sum_{j=1}^{6}\Phi_{N,N}(\boldsymbol{\delta}_j^1), \quad \Phi_{B,B}(\boldsymbol{0}) = -\sum_{j=1}^{3}\Phi_{B,N}(\boldsymbol{\delta}_j^0) - \sum_{j=1}^{6}\Phi_{B,B}(\boldsymbol{\delta}_j^1). \tag{61}$$

Using these FCs, the dynamical matrix can be easily calculated as

$$\begin{aligned} D_{\alpha,\alpha}(\boldsymbol{q}) &= \frac{1}{M_\alpha}\left[\Phi_{\alpha,\alpha}(\boldsymbol{0}) + \sum_{j=1}^{6}\Phi_{\alpha,\alpha}(\boldsymbol{\delta}_j^1)e^{-i\boldsymbol{q}\cdot\boldsymbol{\delta}_j^1}\right], \quad \alpha = N, B, \\ D_{N,B}(\boldsymbol{q}) &= \frac{1}{\sqrt{M_N M_B}}\sum_{j=1}^{3}\Phi_{N,B}(\boldsymbol{\delta}_j^0)e^{-i\boldsymbol{q}\cdot\boldsymbol{\delta}_j^0}, \quad D_{B,N}(\boldsymbol{q}) = D_{N,B}^\dagger(\boldsymbol{q}), \end{aligned} \tag{62}$$

where the mass of atoms is $M_N = 14.0067$ amu and $M_B = 10.811$ amu. The constants above are found to be (unit: $\text{eV} \cdot \text{Å}^{-2}$)

$$t_{xx}^0 = -6.8033, \quad t_{yy}^0 = -33.8892, \quad t_{xx}^1 = -1.6156, \quad t_{xy}^1 = -1.4759, \quad t_{yy}^1 = 0.2661. \tag{63}$$

These values are obtain by MD simulations (Sec. 6).

If the lattice is rotated anti-clockwise by $\theta_l = (-1)^l\theta/2$ ($l = 1, 2$ denote the two layers in twisted bilayer hBN), the intra-layer dynamical matrix $D_{l\alpha,l\beta}^0$ will be rotated from Eq. (62) correspondingly through

$$D_{l\alpha,l\beta}^0(\boldsymbol{q}) = C_{\theta_l}D_{\alpha,\beta}(C_{\theta_l}^{-1}\boldsymbol{q})C_{\theta_l}^{-1}, \quad C_{\theta_l} = \begin{pmatrix} \cos\theta_l & -\sin\theta_l \\ \sin\theta_l & \cos\theta_l \end{pmatrix}. \tag{64}$$

3 Lattice model of 2D PhP in moiré polar systems

3.1 Equation of motion and polarization in moiré superlattice

Moiré systems have a huge sublattice degree of freedom. Here we study modes of moiré materials with atomic thickness (not the setup in Ref. [9]). We use the notations defined in Refs. [10, 11]. Suppose each

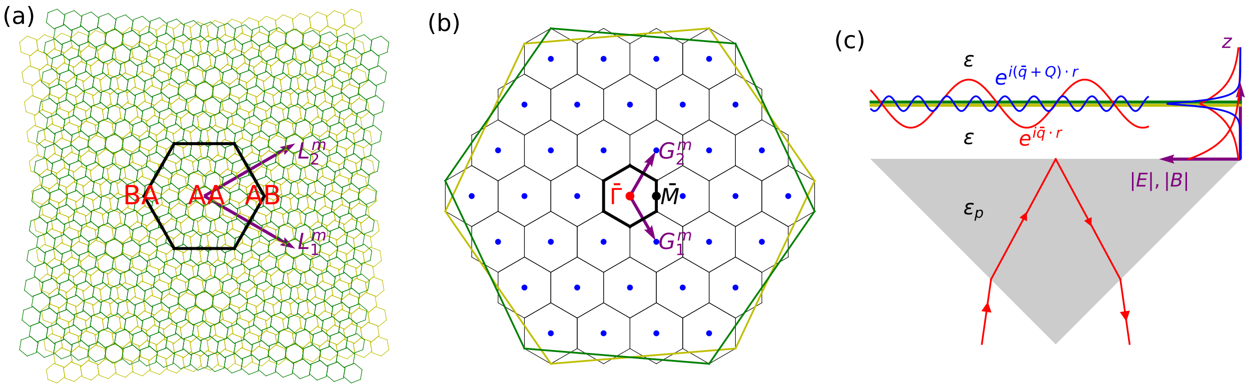

Figure S1: (a) The lattice setup of a twisted bilayer hexagonal lattice. The moiré superlattice is characterized by the supercell (black) and the translation vectors $\boldsymbol{L}^m_{1,2}$. Different stacking regions such AA and AB/BA are also formed. (b) The formation of the corresponding mBZ (bold black) and reciprocal basis vectors $\boldsymbol{G}^m_{1,2}$. The red dot denotes $\bar{\Gamma} = \boldsymbol{0}$. Blue dots denote all other $\boldsymbol{Q} \neq \boldsymbol{0}$ points, which fold into the $\bar{\Gamma}$ point in the mBZ. For visual clarity, in (a) and (b) we plot the case with $N_a = 37$ and $\theta = 9.43°$. (c) The Otto configuration for exciting moiré PhPs in a far-field technique. A prism (gray) is used to generate long-wavelength attenuate incident light (red arrows), which can excite phonons and EM components with shorter wavelengths ($\boldsymbol{Q} \neq \boldsymbol{0}$, blue).

moiré supercell contains N_a atomic unit cells, i.e., the supercell area Ω_m and atomic cell area Ω_0 are related by $\Omega_m = N_a\Omega_0$. Each atom's equilibrium position can be labeled as $\boldsymbol{r}_{Ii\alpha} = \boldsymbol{L}_I + \boldsymbol{R}_i + \boldsymbol{\tau}_\alpha$, where $\boldsymbol{L}_I$ is the moiré Bravais lattice vector, $\boldsymbol{R}_i$ (be careful, $i = 1, ..., N_a$ take finite positions now within a supercell) denotes the atomic Bravais lattice modulo the moiré lattice, and $\boldsymbol{\tau}_\alpha$ is the ion position within each atomic cell. We use $\bar{\boldsymbol{q}}$ to denote wave vectors in the moiré Brillouin zone (mBZ), and $\boldsymbol{Q}$ the moiré reciprocal basis vectors (within the atomic Brillouin zone (aBZ), thus there are totally N_a different $\boldsymbol{Q}$ vectors [11]).

We are interested in the regime with length scale orders

$$a_0 \ll L_\theta \ll \frac{c}{\omega_0}, \tag{65}$$

where a_0, L_θ are atomic and supercell lengths, respectively, and ω_0 is the optical phonon frequency. The moiré potential, here the local force field $\Phi_{i\alpha,j\beta}$, couples different $\boldsymbol{Q}$ components (with length scale L_θ) together. So we insert a multi-wavelength in-plane field at $z = 0$,

$$\boldsymbol{E}_t = \sum_{\boldsymbol{Q}} \boldsymbol{E}_{\bar{\boldsymbol{q}}+\boldsymbol{Q},t} e^{i(\bar{\boldsymbol{q}}+\boldsymbol{Q})\cdot\boldsymbol{r}-i\omega t}, \tag{66}$$

into the equation of motion of the ionic displacement $\boldsymbol{u}$,

$$M_\alpha \ddot{u}_\mu(\boldsymbol{r}_{Ii\alpha}) + \sum_{Jj\beta\nu} \Phi_{i\alpha\mu,j\beta\nu}(\boldsymbol{r}_{Ii\alpha} - \boldsymbol{r}_{Jj\beta}) u_\nu(\boldsymbol{r}_{Jj\beta}) - \sum_{\boldsymbol{Q}} Z_\alpha e E_{\bar{\boldsymbol{q}}+\boldsymbol{Q},\mu}(\omega) e^{i(\bar{\boldsymbol{q}}+\boldsymbol{Q})\cdot\boldsymbol{r}_{Ii\alpha}-i\omega t} = 0. \tag{67}$$

The polarization density is similarly defined as

$$\boldsymbol{P}(\boldsymbol{r}) = \sum_{Ii\alpha} Z_\alpha e \boldsymbol{u}(\boldsymbol{r}_{Ii\alpha}) \delta(\boldsymbol{r} - \boldsymbol{r}_{Ii\alpha}). \tag{68}$$

Even in the present moiré case, the solution is still analytical, and the derivation is almost parallel to the moiré-less case. Suppose $\boldsymbol{u}$ can be expanded as

$$\boldsymbol{u}(\boldsymbol{r}_{Ii\alpha}) = \sum_b \frac{1}{\sqrt{M_\alpha}} e^{i\bar{\boldsymbol{q}}\cdot\boldsymbol{r}_{Ii\alpha}-i\omega t} \boldsymbol{e}_{i\alpha,b}(\bar{\boldsymbol{q}}) B_b(\bar{\boldsymbol{q}}, \omega), \tag{69}$$

where $\boldsymbol{e}_b(\bar{\boldsymbol{q}})$ is the eigenvector corresponding to the b-th field-free mode with frequency $\omega_b(\bar{\boldsymbol{q}})$,

$$\sum_{j\beta\nu} D_{i\alpha\mu,j\beta\nu}(\bar{\boldsymbol{q}})e_{j\beta\nu,b}(\bar{\boldsymbol{q}}) = \omega_b^2(\bar{\boldsymbol{q}})e_{i\alpha\mu,b}(\bar{\boldsymbol{q}}), \tag{70}$$

and the moiré dynamical matrix reads

$$D_{i\alpha,j\beta}(\bar{\boldsymbol{q}}) = \sum_J \frac{\Phi_{i\alpha,j\beta}(\boldsymbol{r}_{I i\alpha} - \boldsymbol{r}_{Jj\beta})}{\sqrt{M_\alpha M_\beta}} e^{i\bar{\boldsymbol{q}}\cdot(\boldsymbol{r}_{Jj\beta}-\boldsymbol{r}_{Ii\alpha})}. \tag{71}$$

The target is to solve for $B_b(\bar{\boldsymbol{q}}, \omega)$. Plugging Eq. (69) into Eq. (67) and using the orthogonality relations

$$\sum_{i\alpha\mu} e^*_{i\alpha\mu,b}(\bar{\boldsymbol{q}})e_{i\alpha\mu,b'}(\bar{\boldsymbol{q}}) = \delta_{bb'}, \quad \sum_b e^*_{i\alpha\mu,b}(\bar{\boldsymbol{q}})e_{j\beta\nu,b}(\bar{\boldsymbol{q}}) = \delta_{ij}\delta_{\alpha\beta}\delta_{\mu\nu}, \tag{72}$$

we get

$$(\omega^2_{\bar{\boldsymbol{q}}b} - \omega^2)B_b(\bar{\boldsymbol{q}}, \omega) = e\sum_{\boldsymbol{Q}} \boldsymbol{S}^*_{\boldsymbol{Q}b}(\bar{\boldsymbol{q}}) \cdot \boldsymbol{E}_{\bar{\boldsymbol{q}}+\boldsymbol{Q},t}, \tag{73}$$

where the moiré S matrix has an additional $\boldsymbol{Q}$ index,

$$\boldsymbol{S}_{\boldsymbol{Q}b}(\bar{\boldsymbol{q}}) = \sum_{i\alpha} \frac{Z_\alpha \boldsymbol{e}_{i\alpha,b}(\bar{\boldsymbol{q}})}{\sqrt{M_\alpha}} e^{-i\boldsymbol{Q}\cdot(\boldsymbol{R}_i+\boldsymbol{\tau}_\alpha)}. \tag{74}$$

We now abstract the continuous field $\boldsymbol{P}(\boldsymbol{r})$ from Eq. (68). Writing Eq. (68) as a Fourier series $\boldsymbol{P}_{\bar{\boldsymbol{q}}+\boldsymbol{Q}}$, but this time we retain some leading terms, i.e., components with $|\boldsymbol{Q}| \ll 2\pi/a_0$ (a_0 is the atomic cell length; higher-order terms are redundant because they detail information within atomic unit cell)

$$\boldsymbol{P}(\boldsymbol{r}) = \sum_{\boldsymbol{Q}} \boldsymbol{P}_{\bar{\boldsymbol{q}}+\boldsymbol{Q}}(\omega)e^{i(\bar{\boldsymbol{q}}+\boldsymbol{Q})\cdot\boldsymbol{r}-i\omega t}. \tag{75}$$

Notice that $\boldsymbol{Q} \neq \boldsymbol{0}$ terms are necessary to retain here because they reflect the dipole fluctuations at moiré length scales. The component $\boldsymbol{P}_{\bar{\boldsymbol{q}}+\boldsymbol{Q}}(\omega)$ is calculated as

$$\boldsymbol{P}_{\bar{\boldsymbol{q}}+\boldsymbol{Q}} = \frac{1}{N_m\Omega_m}\sum_{Ii\alpha} Z_\alpha e\boldsymbol{u}(\boldsymbol{r}_{Ii\alpha})e^{-i(\bar{\boldsymbol{q}}+\boldsymbol{Q})\cdot\boldsymbol{r}_{Ii\alpha}+i\omega t} = \frac{e}{\Omega_m}\sum_b \boldsymbol{S}_{\boldsymbol{Q}b}(\bar{\boldsymbol{q}})B_b(\bar{\boldsymbol{q}}, \omega). \tag{76}$$

Using Eq. (73), we can write

$$P_{\bar{\boldsymbol{q}}+\boldsymbol{Q},\mu}(\omega) = \varepsilon_0 \sum_{\boldsymbol{Q}'\nu} \Pi^{\boldsymbol{Q}\boldsymbol{Q}'}_{\mu\nu}(\bar{\boldsymbol{q}}, \omega)E_{\bar{\boldsymbol{q}}+\boldsymbol{Q}',\nu}, \tag{77}$$

where the susceptibility is now a tensor with index $\boldsymbol{Q}$,

$$\Pi^{\boldsymbol{Q}\boldsymbol{Q}'}_{\mu\nu}(\bar{\boldsymbol{q}}, \omega) = \frac{e^2}{\varepsilon_0\Omega_m}\sum_b \frac{[\boldsymbol{S}_{\boldsymbol{Q}b}(\bar{\boldsymbol{q}})]_\mu[\boldsymbol{S}^*_{\boldsymbol{Q}'b}(\bar{\boldsymbol{q}})]_\nu}{\omega^2_{\bar{\boldsymbol{q}}b} - \omega^2}. \tag{78}$$

It will be convenient to decompose the in-plane fields into directions along and perpendicular to $\bar{\boldsymbol{q}} + \boldsymbol{Q}$,

$$\boldsymbol{E}_{\bar{\boldsymbol{q}}+\boldsymbol{Q},t} = E_{\bar{\boldsymbol{q}}+\boldsymbol{Q},\parallel}\boldsymbol{e}_{\bar{\boldsymbol{q}}+\boldsymbol{Q},\parallel} + E_{\bar{\boldsymbol{q}}+\boldsymbol{Q},\perp}\boldsymbol{e}_{\bar{\boldsymbol{q}}+\boldsymbol{Q},\perp}, \tag{79}$$

where

$$\boldsymbol{e}_{\bar{\boldsymbol{q}}+\boldsymbol{Q},\parallel} = (\bar{\boldsymbol{q}} + \boldsymbol{Q})/|\bar{\boldsymbol{q}} + \boldsymbol{Q}|, \quad \boldsymbol{e}_{\bar{\boldsymbol{q}}+\boldsymbol{Q},\perp} = \boldsymbol{e}_z \times \boldsymbol{e}_{\bar{\boldsymbol{q}}+\boldsymbol{Q},\parallel}. \tag{80}$$

Correspondingly, we can decompose $\Pi^{\boldsymbol{Q}\boldsymbol{Q}'}$ into ($\alpha, \beta = \parallel, \perp$; $\mu, \nu = x, y$)

$$\Pi^{\boldsymbol{Q}\boldsymbol{Q}'}_{\mu\nu}(\bar{\boldsymbol{q}}, \omega) = \sum_{\alpha\beta} \Pi^{\boldsymbol{Q}\boldsymbol{Q}'}_{\alpha\beta}(\bar{\boldsymbol{q}}, \omega)[\boldsymbol{e}_{\bar{\boldsymbol{q}}+\boldsymbol{Q},\alpha}]_\mu[\boldsymbol{e}_{\bar{\boldsymbol{q}}+\boldsymbol{Q}',\beta}]_\nu. \tag{81}$$

The moiré physics enters polaritons by providing the susceptibility $\Pi(\bar{\boldsymbol{q}},\omega)$ with off-diagonal $\boldsymbol{Q}\neq\boldsymbol{Q}'$ terms. In real space, this corresponds to an inhomogeneous optical response. If we turn off the moiré potential, phonons are simply folded from the moiré-less system. In other words, $\boldsymbol{Q}$ remains a good quantum number, and the mode index $b=(\boldsymbol{Q},a)$, where a is the atomic phonon branch index (for hBN, $a=1,...,6$). In this case, Π becomes diagonal in $\boldsymbol{Q}$, and the diagonal term $\Pi^{\boldsymbol{QQ}}(\bar{\boldsymbol{q}},\omega)$ reduces to the moiré-less one with momentum $\bar{\boldsymbol{q}}+\boldsymbol{Q}$. See subsection 5.4 for details.

We note that, in moiré systems, the optical response is still dominated by the in-plane LO and TO modes. The low-frequency shear and layer-breathing (ZO) modes, while highly sensitive to the moiré stacking configuration, contribute negligibly to the optical response in the mid-infrared regime. This is because, the dominant atomic displacements in ZO modes are out-of-plane. For a thin 2D sheet, the long-range electric field coupling essential for 2D PhP formation is to the *in-plane* component of the polarization. ZO modes generate a minimal in-plane dipole moment and are therefore not optically active for coupling to in-plane EM waves, unlike the in-plane TO and LO modes. Besides, the energy difference $\hbar|\omega_{\mathrm{TO}}-\omega_{\mathrm{ZO}}|$ is vastly larger than the moiré potential scattering strength ($\ll 1$ THz). This makes any significant hybridization between high-frequency optical modes and low-frequency ZO modes through the moiré potential physically implausible.

3.2 Moiré polaritons

After the continuation of $\boldsymbol{P}$, the whole system of equations is completed by applying Maxwell's equations, using the method in Sec. 1.5. The moiré material (assumed to have zero thickness) is placed at $z=0$, and the incident light comes from $z<0$. Assume in general that the incident ($l=i$, $z<0$), reflected ($l=r$, $z<0$), and transmitted ($l=t$, $z>0$) light are

$$\begin{aligned}
\boldsymbol{E}^i(\boldsymbol{r},t) &= \sum_{\boldsymbol{Q}\alpha} E^i_{\bar{\boldsymbol{q}}+\boldsymbol{Q},\alpha}\boldsymbol{e}_{\bar{\boldsymbol{q}}+\boldsymbol{Q},\alpha}e^{i(\bar{\boldsymbol{q}}+\boldsymbol{Q})\cdot\boldsymbol{r}-\lambda_{\bar{\boldsymbol{q}}+\boldsymbol{Q}}z-i\omega t},\\
\boldsymbol{E}^r(\boldsymbol{r},t) &= \sum_{\boldsymbol{Q}\alpha} E^r_{\bar{\boldsymbol{q}}+\boldsymbol{Q},\alpha}\boldsymbol{e}_{\bar{\boldsymbol{q}}+\boldsymbol{Q},\alpha}e^{i(\bar{\boldsymbol{q}}+\boldsymbol{Q})\cdot\boldsymbol{r}+\lambda_{\bar{\boldsymbol{q}}+\boldsymbol{Q}}z-i\omega t},\\
\boldsymbol{E}^t(\boldsymbol{r},t) &= \sum_{\boldsymbol{Q}\alpha} E^t_{\bar{\boldsymbol{q}}+\boldsymbol{Q},\alpha}\boldsymbol{e}_{\bar{\boldsymbol{q}}+\boldsymbol{Q},\alpha}e^{i(\bar{\boldsymbol{q}}+\boldsymbol{Q})\cdot\boldsymbol{r}-\lambda_{\bar{\boldsymbol{q}}+\boldsymbol{Q}}z-i\omega t},
\end{aligned} \tag{82}$$

where α includes $\|,\perp,z$, $\boldsymbol{e}_{\bar{\boldsymbol{q}}+\boldsymbol{Q},z}=\boldsymbol{e}_z$, and

$$\lambda_{\bar{\boldsymbol{q}}+\boldsymbol{Q}} = \begin{cases} -i\sqrt{\varepsilon\frac{\omega^2}{c^2}-|\bar{\boldsymbol{q}}+\boldsymbol{Q}|^2}, & |\bar{\boldsymbol{q}}+\boldsymbol{Q}|^2<\varepsilon\frac{\omega^2}{c^2}\\ \sqrt{|\bar{\boldsymbol{q}}+\boldsymbol{Q}|^2-\varepsilon\frac{\omega^2}{c^2}}, & |\bar{\boldsymbol{q}}+\boldsymbol{Q}|^2>\varepsilon\frac{\omega^2}{c^2}\end{cases}. \tag{83}$$

This satisfies Eq. (9). The divergence law [Eq. (8)] requires

$$E^i_{\bar{\boldsymbol{q}}+\boldsymbol{Q},z}=i\frac{|\bar{\boldsymbol{q}}+\boldsymbol{Q}|}{\lambda_{\bar{\boldsymbol{q}}+\boldsymbol{Q}}}E^i_{\bar{\boldsymbol{q}}+\boldsymbol{Q},\|},\quad E^r_{\bar{\boldsymbol{q}}+\boldsymbol{Q},z}=-i\frac{|\bar{\boldsymbol{q}}+\boldsymbol{Q}|}{\lambda_{\bar{\boldsymbol{q}}+\boldsymbol{Q}}}E^r_{\bar{\boldsymbol{q}}+\boldsymbol{Q},\|},\quad E^t_{\bar{\boldsymbol{q}}+\boldsymbol{Q},z}=i\frac{|\bar{\boldsymbol{q}}+\boldsymbol{Q}|}{\lambda_{\bar{\boldsymbol{q}}+\boldsymbol{Q}}}E^t_{\bar{\boldsymbol{q}}+\boldsymbol{Q},\|}. \tag{84}$$

The four BCs at the material surface then yield the following relations (expressed in terms of electric fields)

$$\boldsymbol{E}^t_{\bar{\boldsymbol{q}}+\boldsymbol{Q},t}=\boldsymbol{E}^i_{\bar{\boldsymbol{q}}+\boldsymbol{Q},t}+\boldsymbol{E}^r_{\bar{\boldsymbol{q}}+\boldsymbol{Q},t}, \tag{85a}$$

$$E^t_{\bar{\boldsymbol{q}}+\boldsymbol{Q},z}-E^i_{\bar{\boldsymbol{q}}+\boldsymbol{Q},z}-E^r_{\bar{\boldsymbol{q}}+\boldsymbol{Q},z}=-\frac{i(\bar{\boldsymbol{q}}+\boldsymbol{Q})\cdot\boldsymbol{P}_{\bar{\boldsymbol{q}}+\boldsymbol{Q}}}{\varepsilon_0\varepsilon}, \tag{85b}$$

$$\lambda_{\bar{\boldsymbol{q}}+\boldsymbol{Q}}(\boldsymbol{E}^i_{\bar{\boldsymbol{q}}+\boldsymbol{Q},t}-\boldsymbol{E}^r_{\bar{\boldsymbol{q}}+\boldsymbol{Q},t}-\boldsymbol{E}^t_{\bar{\boldsymbol{q}}+\boldsymbol{Q},t})-i(\bar{\boldsymbol{q}}+\boldsymbol{Q})(E^t_{\bar{\boldsymbol{q}}+\boldsymbol{Q},z}-E^i_{\bar{\boldsymbol{q}}+\boldsymbol{Q},z}-E^r_{\bar{\boldsymbol{q}}+\boldsymbol{Q},z})=-\mu_0\omega^2\boldsymbol{P}_{\bar{\boldsymbol{q}}+\boldsymbol{Q}}, \tag{85c}$$

$$\boldsymbol{e}_z\cdot\left[(\bar{\boldsymbol{q}}+\boldsymbol{Q})\times(\boldsymbol{E}^t_{\bar{\boldsymbol{q}}+\boldsymbol{Q},\perp}-\boldsymbol{E}^i_{\bar{\boldsymbol{q}}+\boldsymbol{Q},\perp}-\boldsymbol{E}^r_{\bar{\boldsymbol{q}}+\boldsymbol{Q},\perp})\right]=0. \tag{85d}$$

Similar to the toy model case in Sec. 1.1, it is sufficient to consider only the 1st, 2nd, and the transverse components of the 3rd BCs (for each $\boldsymbol{Q}$). The 4th BC and the longitudinal component of the 3rd BC coincide with the 1st and 2nd BCs, respectively. Substituting Eqs. (77) and (84) into the above BCs, and using the 1st BC to eliminate $\boldsymbol{E}^r_t$, we can organize these BCs into a set of linear equations

$$\sum_{\boldsymbol{Q}'\beta}A^{\boldsymbol{QQ}'}_{\alpha\beta}(\bar{\boldsymbol{q}},\omega)\boldsymbol{E}^t_{\bar{\boldsymbol{q}}+\boldsymbol{Q}',\beta}=\boldsymbol{E}^i_{\bar{\boldsymbol{q}}+\boldsymbol{Q},\alpha}, \tag{86}$$

with matrix elements

$$A^{\boldsymbol{Q}\boldsymbol{Q}'}_{\parallel\parallel}(\bar{\boldsymbol{q}},\omega) = \delta_{\boldsymbol{Q}\boldsymbol{Q}'} + \frac{\lambda_{\bar{\boldsymbol{q}}+\boldsymbol{Q}}}{2\varepsilon}\Pi^{\boldsymbol{Q}\boldsymbol{Q}'}_{\parallel\parallel}(\bar{\boldsymbol{q}},\omega), \tag{87a}$$

$$A^{\boldsymbol{Q}\boldsymbol{Q}'}_{\parallel\perp}(\bar{\boldsymbol{q}},\omega) = \frac{\lambda_{\bar{\boldsymbol{q}}+\boldsymbol{Q}}}{2\varepsilon}\Pi^{\boldsymbol{Q}\boldsymbol{Q}'}_{\parallel\perp}(\bar{\boldsymbol{q}},\omega), \tag{87b}$$

$$A^{\boldsymbol{Q}\boldsymbol{Q}'}_{\perp\parallel}(\bar{\boldsymbol{q}},\omega) = -\frac{1}{2\lambda_{\bar{\boldsymbol{q}}+\boldsymbol{Q}}}\frac{\omega^2}{c^2}\Pi^{\boldsymbol{Q}\boldsymbol{Q}'}_{\perp\parallel}(\bar{\boldsymbol{q}},\omega), \tag{87c}$$

$$A^{\boldsymbol{Q}\boldsymbol{Q}'}_{\perp\perp}(\bar{\boldsymbol{q}},\omega) = \delta_{\boldsymbol{Q}\boldsymbol{Q}'} - \frac{1}{2\lambda_{\bar{\boldsymbol{q}}+\boldsymbol{Q}}}\frac{\omega^2}{c^2}\Pi^{\boldsymbol{Q}\boldsymbol{Q}'}_{\perp\perp}(\bar{\boldsymbol{q}},\omega). \tag{87d}$$

The above equation generalizes Eqs. (27), (28), and is a core result of this paper. From Eq. (86), we can define the transmission and reflection tensors

$$T(\bar{\boldsymbol{q}},\omega) = A^{-1}(\bar{\boldsymbol{q}},\omega), \quad R(\bar{\boldsymbol{q}},\omega) = A^{-1}(\bar{\boldsymbol{q}},\omega) - I, \tag{88}$$

such that

$$\boldsymbol{E}^t_t = T(\bar{\boldsymbol{q}},\omega)\boldsymbol{E}^i_t, \quad \boldsymbol{E}^r_t = R(\bar{\boldsymbol{q}},\omega)\boldsymbol{E}^i_t, \tag{89}$$

written in the basis:

$$\boldsymbol{E}^l_t = (E^l_{\bar{\boldsymbol{q}}+\boldsymbol{Q}_1,\parallel}, E^l_{\bar{\boldsymbol{q}}+\boldsymbol{Q}_1,\perp}, ..., E^l_{\bar{\boldsymbol{q}}+\boldsymbol{Q}_{N_a},\perp})^T. \tag{90}$$

All information about PhPs is contained in $A(\bar{\boldsymbol{q}},\omega)$; for example, the zeros of $\det(A)$ determines the polariton dispersion, and the eigenvectors of A correspond to the respective eigenmode EM fields. In matrix form, Eq. (86) is $A\boldsymbol{E}^t = \boldsymbol{E}^i$ or $\boldsymbol{E}^t = A^{-1}\boldsymbol{E}^i$. If the incident light is long-wavelength, i.e., $\boldsymbol{E}^i = (\boldsymbol{E}^i_{\bar{\boldsymbol{q}}}, \boldsymbol{0}, \boldsymbol{0}, ...)$, then because A^{-1} is not diagonal in $\boldsymbol{Q}$ (since A is not diagonal), we generally have $\boldsymbol{E}^t_{\bar{\boldsymbol{q}}+\boldsymbol{Q}} = [A^{-1}]_{\boldsymbol{Q}\boldsymbol{0}}\boldsymbol{E}^i_{\bar{\boldsymbol{q}}} \neq \boldsymbol{0}$. Physically, this means that a long-wavelength incident light could induce a response with short-wavelength components, via scattering by the moiré potential (encoded in the A matrix). This effect, depicted in Fig. S1(c), is a salient feature of moiré PhPs, which never occurs in moiré-less systems.

Based on this property, we can focus on long-wavelength incidence, which already captures information about moiré scattering and excludes short-wavelength contributions. The effective transmission matrix is the $\boldsymbol{Q} = \boldsymbol{0}$ submatrix [12]:

$$T_{\text{eff}}(\bar{\boldsymbol{q}},\omega) = [A^{-1}(\bar{\boldsymbol{q}},\omega)]^{\boldsymbol{0}\boldsymbol{0}}. \tag{91}$$

The poles of the spectrum $\mathcal{L}(\bar{\boldsymbol{q}},\omega) = -\text{Im}[\det[T_{\text{eff}}(\bar{\boldsymbol{q}},\omega+i\delta/2)]]$ describe the dispersion of moiré PhPs that can be excited by long-wavelength light. Since $\mathcal{L}$ can change sign at certain points, we instead plot the spectrum of $\ln(1+|\mathcal{L}(\bar{\boldsymbol{q}},\omega)|)$, whose poles are the same to those of $\mathcal{L}$, to visualize the dispersions of moiré PhPs.

3.3 Moiré PhP dispersion against phonon linewidth

In Fig. 2 of the main text, the PhP dispersion is plotted using an extremely small phonon linewidth $\delta/(2\pi) = 0.001$ THz. Each moiré branch is distinguishable and separate from the others only if such a tiny loss is assumed. However, in realistic physical systems, the phonon linewidth δ is finite. It quantifies the finite lifetime of phonons due to intrinsic (e.g., anharmonic scattering, electron-phonon coupling) or external (e.g., defects, boundaries, radiative sources) mechanisms. In general, δ is momentum- and frequency-dependent, but here we approximate it as constant. Typically, for the optical branches of hBN, the optimized δ ranges between 0.2-0.5 THz [13, 14, 15], which is much larger than the value adopted in Fig. 2 of the main text.

To determine whether the moiré PhP dispersion can survive under realistic conditions, we calculate the PhP spectrum of twisted bilayer 2.65° hBN for different phonon linewidths: 0.001, 0.01, 0.1, and 0.3 THz. The results are shown in Fig. S2. The dispersion is clearly visible for $\delta/(2\pi) \leq 0.01$ THz [Fig. S2(a)(b)], and becomes obscure as δ increases further. In the range of realistic phonon linewidth ($\delta \geq 0.1$ THz), some moiré branches 'merge' with others, and only the rough outlines are recognizable. For $\delta/(2\pi) = 0.1$ THz [Fig. S2(c)], we can still distinguish some moiré branches, though the fine structure shown in Fig. 2(b) of the main text is obscured. For $\delta/(2\pi) = 0.3$ THz [Fig. S2(d)], the spectrum becomes more mixed. In this case, we can hardly distinguish the dispersion of each PhP branch from the spectrum, but we can roughly identify the frequency region (49.1-49.5 THz) where moiré physics dominates.

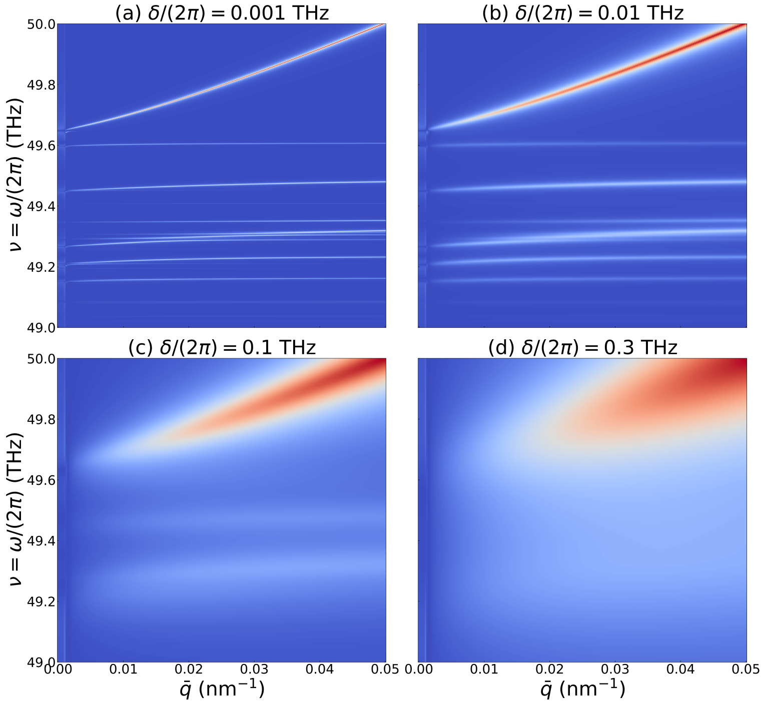

Figure S2: The PhP dispersion of 2.65° twisted bilayer hBN, calculated with the linewidth (a) $\delta/(2\pi) = 0.001$ THz, (b) $\delta/(2\pi) = 0.01$ THz, (c) $\delta/(2\pi) = 0.1$ THz, and (d) $\delta/(2\pi) = 0.3$ THz.

We find that the PhP spectrum depends quite sensitively on δ in the realistic range. The moiré PhP dispersion can be captured only in extremely optimized samples with sufficiently low loss (e.g., $\delta \approx 0.1$ THz). Thus, the direct detection of PhP dispersion using traditional far-field setups is challenging. Instead, as shown in Figs. 3 and 4 of the main text, electric fields fluctuate in real space at specific frequencies, proving a more compelling signal to verify the existence of moiré PhPs.

3.4 PhP spectrum of twisted bilayer MoTe2 system

In $MoTe_2$, the ions carry effective charges $Z_{\mathrm{Mo}} = -2Z_{\mathrm{Te}} \approx 3.16$ (in units of e, obtained from DFT calculations), and the ionic masses are larger than those of hBN. The critical optical phonon frequency is $\omega_0/(2\pi) \approx 7.2$ THz. For twisted bilayer systems, the force constant model can be constructed using MD simulations (Section 6).

We have calculated the PhP spectrum of 3.89° twisted bilayer $MoTe_2$, which has aroused wide interest due to the discovery of its fractional Chern insulating phase. The result is shown in Fig. S3(a). Comparing it with hBN (Fig. 2 of the main text), we find the qualitative structures of the PhP dispersion are the same. For example, the PhP dispersions consist of some mini-branches and a dominant branch. The mini-branches have smaller bandwidths and intensities, encoding mainly moiré components, while the dominant branch has a linear slope and stronger intensity, inheriting mainly from the moiré-less TM mode. Additionally, the intensities of the mini-branches weaken as ω moves far from ω_0.

We then analyze some differences with hBN, most of which are quantitative. Both the critical frequency ω_0 and the mini-band gaps (at $\bar{\boldsymbol{q}} = \mathbf{0}$, ≈ 0.01 THz) are smaller than those in hBN. This is mainly due to the heavier mass in $MoTe_2$. The number of mini-branches is smaller than in hBN, because the 3.89° system has a much smaller supercell size than the 2.65° supercell in hBN. More moiré PhP branches (and smaller band gaps) are expected with a smaller twisting angle.

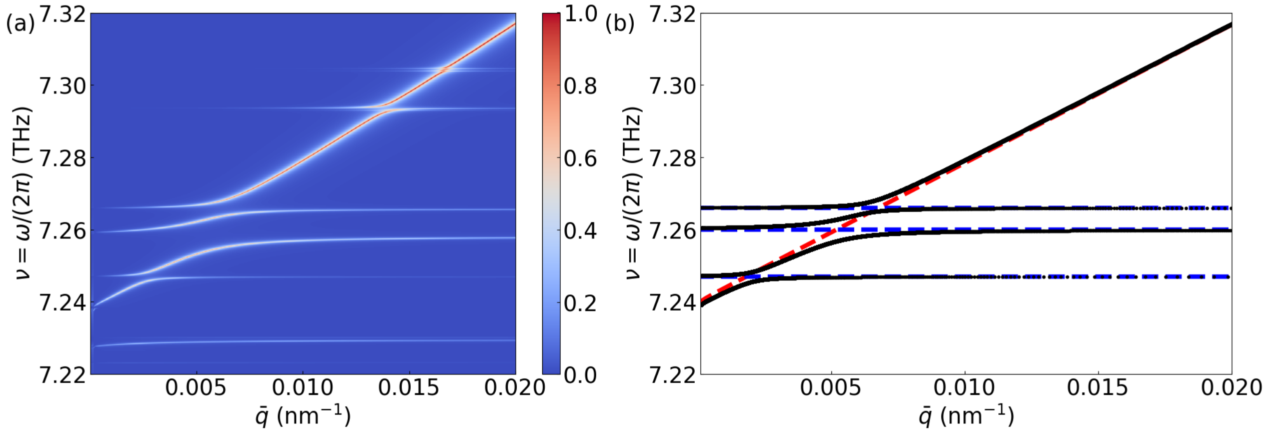

Figure S3: (a) Long-wavelength PhP dispersion (along the $\bar{\Gamma} - \bar{M}$ direction) for 3.89° twisted bilayer $MoTe_2$, near the frequency $\nu_0 \approx 7.2$ THz ($q_0 \approx 1.5 \times 10^{-4}$ nm^{-1}), calculated from the lattice model. Here we take $\delta/2\pi = 2 \times 10^{-4}$ THz. (b) The same PhP dispersion (black) obtained using the coupled oscillator model (TM mode). Here we take $\gamma_0^2/(2\pi)^4 = 3.9 \times 10^7$ CV^{-1}s^{-2}, and for $\mathcal{D}$ parameters [in the unit of $(2\pi\text{THz})^2$]: $\mathcal{D}_{00} = 7.240^2$, $\mathcal{D}_{11} = 7.247^2$, $\mathcal{D}_{22} = 7.260^2$, $\mathcal{D}_{33} = 7.266^2$, $\mathcal{D}_{01} = \mathcal{D}_{10} = 0.0204$, $\mathcal{D}_{02} = \mathcal{D}_{20} = 0.0456$, $\mathcal{D}_{03} = \mathcal{D}_{30} = 0.0236$, (all other parameters are set to zero), obtained by fitting (a). For comparison, we also show the PhP dispersion (red dashed line) and the high-momentum $\boldsymbol{Q}$ phonon frequencies (blue dashed lines) for the decoupled case.

4 Macroscopic theory of moiré PhP

4.1 A toy model: coupled harmonic oscillators

In the presence of inhomogeneity, the system is described by a set of coupled vibration fields $\boldsymbol{W}_a$ and electric fields $\boldsymbol{E}_a$. In the present case a labels just the different Fourier components $\boldsymbol{Q}$. More specifically, we use $\boldsymbol{W}_0$ and $\boldsymbol{E}_0$ to denote the usual long-wavelength fields (with momentum $\bar{\boldsymbol{q}} \approx \boldsymbol{0}$), while all other components ($a \neq 0$) correspond to high-momentum fields (with momentum $\bar{\boldsymbol{q}} + \boldsymbol{Q}$). The equation of motion and polarization density for the a-th component are

$$\ddot{\boldsymbol{W}}_a = -\sum_{a'} \mathcal{D}_{aa'} \boldsymbol{W}_{a'} + \gamma \boldsymbol{E}_a, \quad \boldsymbol{P}_a = \gamma \boldsymbol{W}_a, \tag{92}$$

where $\mathcal{D}_{aa'} = D^*_{a'a}$ is the coupling constant among $\boldsymbol{W}_a$ and $\boldsymbol{W}_{a'}$, γ is the charge coefficient. Notice that $\mathcal{D}_{aa} = \omega_a^2$ is the elastic eigenfrequency. The nontrivial inhomogeneity leads to $\mathcal{D}_{aa'} \neq 0$ when $a \neq a'$. We can decompose $\boldsymbol{W}_a$ into normal modes $\boldsymbol{W}_a = \sum_b U_{ab} \boldsymbol{w}_b$ that diagonalize the coupled system, where the orthogonal transformation matrix V satisfies

$$\sum_a (\omega_b^2 \delta_{a'a} - \mathcal{D}_{a'a}) U_{ab} = 0, \quad \sum_a U^*_{ab} U_{ab'} = \delta_{bb'}, \quad \sum_b U_{ab} U^*_{a'b} = \delta_{aa'}. \tag{93}$$

One can show that (for oscillation at frequency ω)

$$\ddot{\boldsymbol{w}}_b = -\omega_b^2 \boldsymbol{w}_b + \gamma \sum_a U^*_{ab} \boldsymbol{E}_a, \quad \boldsymbol{P}_a = \gamma \sum_b U_{ab} \boldsymbol{w}_b = \sum_{a'} \sum_b \gamma^2 \frac{U_{ab} U^*_{a'b}}{\omega_b^2 - \omega^2} \boldsymbol{E}_{a'}. \tag{94}$$

If we focus on the long-wavelength dispersion, we may neglect all short-wavelength fields $\boldsymbol{w}_b$, $\boldsymbol{E}_b$, and $\boldsymbol{P}_b$ with $b \neq 0$. If so, after redefining $T_b = |\gamma U_{0b}|^2/\varepsilon_0$, we obtain the effective long-wavelength susceptibility

$$\Pi(\omega) = \Pi^{00}(\omega) = \sum_b \frac{T_b}{\omega_b^2 - \omega^2}, \tag{95}$$

which has multiple poles. By plugging this long-wavelength susceptibility into Eq. (19), we can obtain the moiré PhP dispersion. In Fig. S3 we plot the TM dispersion using this method and fitting the model of

twisted $MoTe_2$. Notice that each pole ω_b has a specific strength T_b, proportional to the scattering strength into that channel, and gives rise to a specific pair of TM and TE modes in the absence of other poles. The TE modes are mainly lattice vibrations, contributing little to the PhP dispersion. The TM modes disperse linearly with slopes proportional to T_b starting from ω_b [Eq. (13)]. Therefore, crossings occur between these branches with different slopes if they are not coupled. The whole frequency region is partitioned into a series of negative windows where $\Pi(\omega) < 0$ and positive windows where $\Pi(\omega) > 0$. Remembering the sign rule, the TM (TE) PhP lies separately in the negative (positive) window. This is true even when all the poles are taken into consideration. This results in the anti-crossings shown in Fig. S3(b). The most dispersive (dominant) branch has the largest T_b, which carries the most long-wavelength component. The moiré pattern of $\boldsymbol{E}$ and $\boldsymbol{W}$ is a byproduct: when ω approaches ω_b, the lattice oscillates exclusively in accordance with mode b, which generates a short-wavelength polarization, thus jointly inducing the moiré EM fields.

We observe that Ref. [16] adopts a similar model to explain the exciton polariton. Their two poles originate from the heterostructure. Here, the multiple poles result from umklapp scattering due to the inhomogeneous moiré potential in the homobilayer. This is also why these poles ω_b are so close to each other. The coupled oscillator model presented in this subsection is only a toy model. It is only used to illustrate how the moiré potential produces multiple branches of PhP. We will refine this model in the next subsection to make it more quantitative and accurate.

4.2 The continuum model for moiré phonon

A more accurate model should incorporate the anisotropy of TO and LO phonons at various moiré reciprocal vectors $\boldsymbol{Q}$. The macroscopic model for moiré PhP requires a corresponding continuum model for optical moiré phonons (without long-wavelength electric field). The theoretical structure is quite similar to the Bistritzer-MacDonald (BM) model for electrons in the magic-angle twisted bilayer graphene [17], and is more conveniently obtained using the truncated plane atomic wave (TAPW) method [10, 11].

The dynamical matrix (71) has exactly the same form as the tight-binding Hamiltonian for electrons, thus suggesting the same mathematical and physical structure. The general idea of the TAPW method is to expand the eigenmodes of the moiré system using the monolayer "plane waves", i.e.,

$$e_{i\alpha\mu,b}(\bar{\boldsymbol{k}}) = \sum_{\boldsymbol{Q}la} \frac{e^{i\boldsymbol{Q}\cdot(\boldsymbol{R}_i+\boldsymbol{\tau}_\alpha)}}{\sqrt{N_a}} e_{\alpha\mu,la}(\bar{\boldsymbol{q}}+\boldsymbol{Q}) U_{\boldsymbol{Q}la,b}(\bar{\boldsymbol{q}}), \tag{96}$$

where the vector $\boldsymbol{e}_{la}(\boldsymbol{q})$ (a: branch, l: layer) satisfies the monolayer dynamical equation Eq. (35) and orthogonality Eq. (36). If we turn off the moiré potential, $U(\bar{\boldsymbol{q}})$ becomes diagonal and the phonons simply reduce to those folded from the monolayer case without any hybridization (Sec. 5.4). We can expect that a small truncation of $\boldsymbol{Q}$ vectors in the Γ valley (here we take $N_{\boldsymbol{Q}} = 61$) is sufficient to calculate the phonons there. Since we only focus on the iLO and iTO modes, we limit the summation over a to these two branches. Therefore, the TAPW method reduces the dimension to $4N_{\boldsymbol{Q}}$. The U matrix satisfies

$$\sum_{\boldsymbol{Q}'l'a'} \mathcal{D}_{\boldsymbol{Q}la,\boldsymbol{Q}'l'a'}(\bar{\boldsymbol{q}}) U_{\boldsymbol{Q}'l'a',b}(\bar{\boldsymbol{q}}) = \omega^2_{\bar{\boldsymbol{q}}b} U_{\boldsymbol{Q}la,b}(\bar{\boldsymbol{q}}), \tag{97}$$

where the transformed dynamical matrix is derived to be [10, 11]

$$\mathcal{D}_{\boldsymbol{Q}la,\boldsymbol{Q}'l'a'}(\bar{\boldsymbol{q}}) = \sum_{i\alpha\mu,j\beta\nu} e^*_{\alpha\mu,la}(\bar{\boldsymbol{q}}+\boldsymbol{Q}) \frac{e^{-i\boldsymbol{Q}\cdot(\boldsymbol{R}_i+\boldsymbol{\tau}_\alpha)}}{\sqrt{N_a}} D_{i\alpha\mu,j\beta\nu}(\bar{\boldsymbol{q}}) \frac{e^{i\boldsymbol{Q}'\cdot(\boldsymbol{R}_j+\boldsymbol{\tau}_\beta)}}{\sqrt{N_a}} e_{\beta\nu,l'a'}(\bar{\boldsymbol{q}}+\boldsymbol{Q}'). \tag{98}$$

Our next goal is to simplify $\mathcal{D}(\bar{\boldsymbol{q}})$. For convenience, we fix the direction of iLO/iTO basis vectors at different $\bar{\boldsymbol{q}}+\boldsymbol{Q}$, and use the following basis at $\boldsymbol{q}=\boldsymbol{0}$ to replace $\boldsymbol{e}_{\alpha,la}(\boldsymbol{q})$ [see Eq. (50)]

$$\boldsymbol{e}_{lx} = \left(\sqrt{\frac{M_B}{M_N+M_B}}, 0, -\sqrt{\frac{M_N}{M_N+M_B}}, 0\right)^T, \quad \boldsymbol{e}_{ly} = \left(0, \sqrt{\frac{M_B}{M_N+M_B}}, 0, -\sqrt{\frac{M_N}{M_N+M_B}}\right)^T. \tag{99}$$

This step itself is an approximation that assumes the Hilbert space spanned by iLO/iTO modes at $\bar{\boldsymbol{q}}+\boldsymbol{Q}$ is the same as the Γ point. But it greatly simplifies the calculation since it avoids the complicated $\boldsymbol{q}$-dependence of $\boldsymbol{e}_{la}(\boldsymbol{q})$ in the spirit of $k\cdot p$ theory. Correspondingly, we replace the branch index a =iLO,iTO with the index

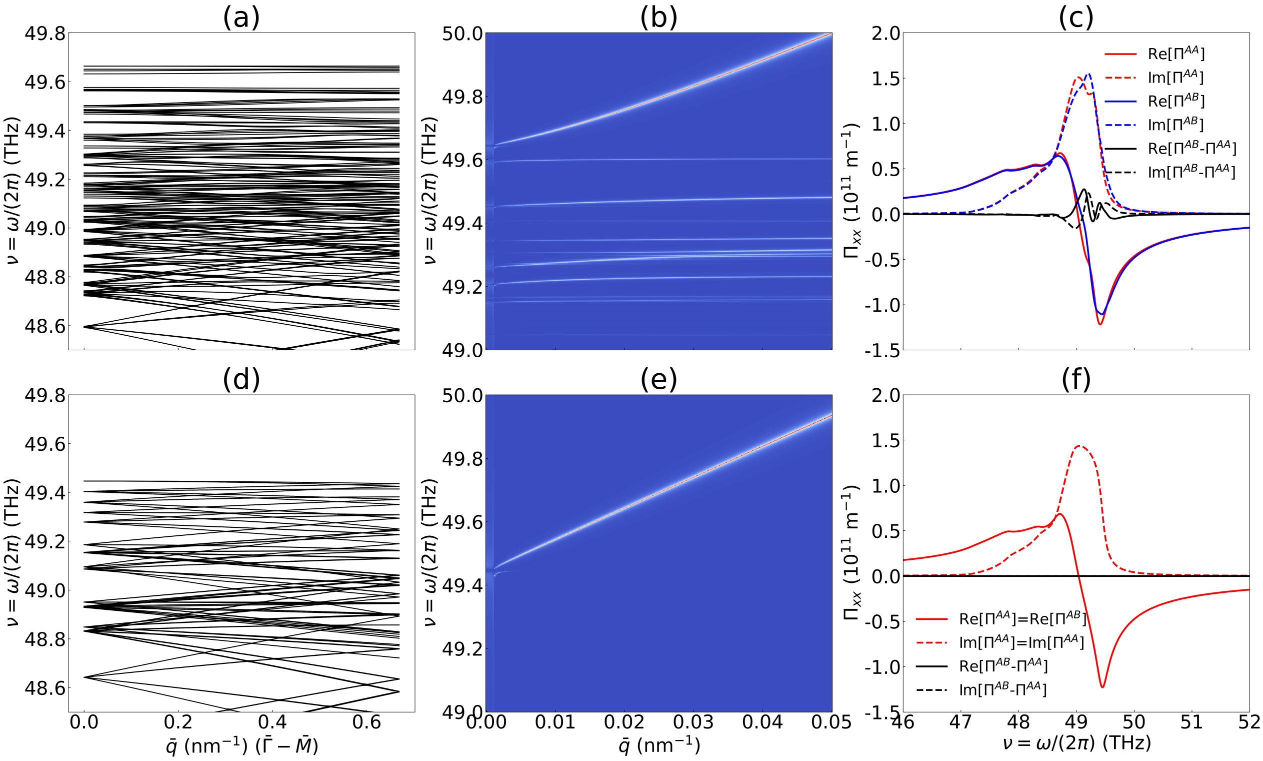

Figure S4: Phonon bands [(a), (d)], PhP bands [(b), (e)], and the local susceptibility $\Pi_{xx}(\boldsymbol{r}, \boldsymbol{r}, \omega)$ [(c), (f)], calculated using the continuum model from Secs. 4.2 and 4.3. (a), (b), (c) show results for 2.65° twisted bilayer hBN, while in (d), (e), (f) we have turned off the moiré potential, i.e., we set $\delta\mathcal{D}^{\text{intra}} = \delta\mathcal{D}^{\text{inter}} = 0$. All calculations use a basis of 61 $\boldsymbol{Q}$ vectors. In (b), (e) we use the linewidth $\delta/(2\pi) = 0.001$ THz. In (c), (f) we use $\delta/(2\pi) = 0.15$ THz.

$\mu = x, y$. Note that here μ indexes the mode, not the spatial direction. The dynamical matrix in the basis of Eq. (99) then becomes

$$\mathcal{D}_{\boldsymbol{Q}l\mu,\boldsymbol{Q}'l'\nu}(\bar{\boldsymbol{q}}) = \sum_{i\alpha,j\beta} \frac{e^{-i\boldsymbol{Q}\cdot(\boldsymbol{R}_i+\boldsymbol{\tau}_\alpha)}}{\sqrt{N_a}} \boldsymbol{e}^*_{\alpha,l\mu} D_{i\alpha,j\beta}(\bar{\boldsymbol{q}}) \boldsymbol{e}_{\beta,l'\nu} \frac{e^{i\boldsymbol{Q}'\cdot(\boldsymbol{R}_j+\boldsymbol{\tau}_\beta)}}{\sqrt{N_a}}. \tag{100}$$

The intralayer $l = l'$ terms with $\boldsymbol{Q} = \boldsymbol{Q}'$ are straightforward to evaluate. We can simply diagonalize the monolayer dynamical matrix at $\bar{\boldsymbol{q}} + \boldsymbol{Q}$ [i.e., $D^0_{l\alpha,l\beta}(\bar{\boldsymbol{q}} + \boldsymbol{Q})$ defined in Eq. (64)], and use the monolayer iLO/iTO frequencies (denoted by $\omega^0_{\bar{\boldsymbol{q}}+\boldsymbol{Q},la}$) to write

$$\mathcal{D}^0_{\boldsymbol{Q}l\mu,\boldsymbol{Q}l\nu}(\bar{\boldsymbol{q}}) = \sum_a^{\text{iLO,iTO}} (\omega^0_{\bar{\boldsymbol{q}}+\boldsymbol{Q},la})^2 \boldsymbol{e}^T_{l\mu} \boldsymbol{e}_{la}(\bar{\boldsymbol{q}} + \boldsymbol{Q}) \boldsymbol{e}^T_{la}(\bar{\boldsymbol{q}} + \boldsymbol{Q}) \boldsymbol{e}_{l\nu}, \tag{101}$$

where $\boldsymbol{e}_{la}(\boldsymbol{q})$ is defined in Eq. (49). We now introduce the moiré potential. The moiré potential generally consists of two parts: an intralayer part, arising primarily from lattice relaxation, and an interlayer part, due mainly to commensurate interlayer scattering. For simplicity, we retain only the hoppings among nearest $\boldsymbol{Q}$ vectors (just like the BM model). The intralayer moiré potential can then be expressed in the $\bar{\boldsymbol{q}}$-independent form

$$\delta\mathcal{D}^{\text{intra}}_{\boldsymbol{Q}l\mu,\boldsymbol{Q}'l\nu} = \sum_{j=1,2,3} \left[B_{l\mu,l\nu}(\boldsymbol{G}^m_j)\delta_{\boldsymbol{Q},\boldsymbol{Q}'+\boldsymbol{G}^m_j} + B^*_{l\mu,l\nu}(\boldsymbol{G}^m_j)\delta_{\boldsymbol{Q},\boldsymbol{Q}'-\boldsymbol{G}^m_j} \right], \tag{102}$$

where $\boldsymbol{G}^m_1 = 4\pi/(\sqrt{3}L_\theta)(1/2, -\sqrt{3}/2)$, $\boldsymbol{G}^m_2 = C_{3z}\boldsymbol{G}^m_1$, $\boldsymbol{G}^m_3 = C^{-1}_{3z}\boldsymbol{G}^m_1$, L_θ is the moiré supercell length. The B matrix with argument $\boldsymbol{G}^m_1$ is found to be

$$B_{1,1}(\boldsymbol{G}^m_1) = \begin{pmatrix} 256.20 - 3.09i & -161.46 - 4.68i \\ -161.46 - 4.68i & -292.64 - 2.84i \end{pmatrix}, \tag{103}$$

for 2.65° bilayer hBN. The numbers here (and below) are in the unit of THz^2. The interlayer part is found to be much smaller than the intralayer part. It is given by ($\bar{l}$ denotes another layer of l)

$$\delta\mathcal{D}^{\text{inter}}_{\boldsymbol{Q}l\mu,\boldsymbol{Q}'\bar{l}\nu} = B_{l\mu,\bar{l}\nu}(\boldsymbol{0})\delta_{\boldsymbol{Q},\boldsymbol{Q}'} + \sum_{j=1,2,3} \left[B_{l\mu,\bar{l}\nu}(\boldsymbol{G}^m_j)\delta_{\boldsymbol{Q},\boldsymbol{Q}'+\boldsymbol{G}^m_j} + B^*_{l\mu,\bar{l}\nu}(\boldsymbol{G}^m_j)\delta_{\boldsymbol{Q},\boldsymbol{Q}'-\boldsymbol{G}^m_j} \right], \tag{104}$$

with

$$B_{1,2}(\boldsymbol{0}) = \begin{pmatrix} -7.64 & \\ & -7.64 \end{pmatrix}, \quad B_{1,2}(\boldsymbol{G}^m_1) = \begin{pmatrix} 9.20 & 6.74 \\ 6.74 & 1.41 \end{pmatrix}. \tag{105}$$

The other B matrices can be obtained through time reversal $\mathcal{T}$, C_{3z} and C_{2y} rotations:

$$\begin{aligned} B_{\bar{l},\bar{l}'}(\boldsymbol{G}^m_1) &= C_{2y}C_{3z}B^*_{l,l'}(\boldsymbol{G}^m_1)C^T_{3z}C_{2y}, \\ B_{l,l'}(\boldsymbol{G}^m_2) &= C_{3z}B_{l,l'}(\boldsymbol{G}^m_1)C^T_{3z}, \\ B_{l,l'}(\boldsymbol{G}^m_3) &= C^T_{3z}B_{l,l'}(\boldsymbol{G}^m_1)C_{3z}. \end{aligned} \tag{106}$$

By treating the phonon fields as continuum plane waves indexed by layer and sublattice, the total dynamical matrix can be written in a more compact form that resembles the BM Hamiltonian,

$$D(-i\nabla, \boldsymbol{r}) = \begin{pmatrix} \mathcal{D}^0_1(-i\nabla) + \delta\mathcal{D}^{\text{intra}}_{1,1}(\boldsymbol{r}) & \delta\mathcal{D}^{\text{inter}}_{1,2}(\boldsymbol{r}) \\ \delta\mathcal{D}^{\text{inter}}_{2,1}(\boldsymbol{r}) & \mathcal{D}^0_2(-i\nabla) + \delta\mathcal{D}^{\text{intra}}_{2,2}(\boldsymbol{r}) \end{pmatrix}, \tag{107}$$

where $\mathcal{D}^0_l(-i\nabla)$ represents the iLO/iTO frequencies and is diagonal in $\boldsymbol{Q}$ with matrix element given by Eq. (101), and

$$\begin{aligned} \delta\mathcal{D}^{\text{intra}}_{l,l}(\boldsymbol{r}) &= \sum_{j=1,2,3} B_{l,l}(\boldsymbol{G}^m_j)e^{i\boldsymbol{G}^m_j\cdot\boldsymbol{r}} + h.c., \\ \delta\mathcal{D}^{\text{inter}}_{l,\bar{l}}(\boldsymbol{r}) &= \frac{1}{2}B_{lA,\bar{l}A'}(\boldsymbol{0}) + \sum_{j=1,2,3} B_{l,\bar{l}}(\boldsymbol{G}^m_j)e^{i\boldsymbol{G}^m_j\cdot\boldsymbol{r}} + h.c.. \end{aligned} \tag{108}$$

4.3 The continuum model for moiré PhP

The continuum model for moiré PhP can be easily obtained through the moiré phonon model introduced in the last subsection. Firstly, we notice that in moiré systems, the continuum version of displacement field $\boldsymbol{u}_{l\alpha}$ can be written as

$$\boldsymbol{u}_{l\alpha}(\boldsymbol{r},t) = \sum_{\boldsymbol{Q}} \boldsymbol{u}_{l\alpha,\bar{\boldsymbol{q}}+\boldsymbol{Q}}(\boldsymbol{r},t), \tag{109}$$

where $\boldsymbol{u}_{l\alpha,\bar{\boldsymbol{q}}+\boldsymbol{Q}} \propto e^{i(\bar{\boldsymbol{q}}+\boldsymbol{Q})\cdot\boldsymbol{r}}$ is the $\boldsymbol{Q}$-th Fourier component of $\boldsymbol{u}_{l\alpha}$ in layer l. Like Eq. (53), we define a series of continuum fields, characterized by the wavevector $\boldsymbol{Q}$, at layer l,

$$\boldsymbol{W}_{\bar{\boldsymbol{q}}+\boldsymbol{Q},l}(\boldsymbol{r},t) = \frac{1}{\sqrt{\Omega_0}}\sqrt{\frac{M_N M_B}{M_N+M_B}}[\boldsymbol{u}_{lN,\bar{\boldsymbol{q}}+\boldsymbol{Q}}(\boldsymbol{r},t) - \boldsymbol{u}_{lB,\bar{\boldsymbol{q}}+\boldsymbol{Q}}(\boldsymbol{r},t)] = \boldsymbol{W}_{\bar{\boldsymbol{q}}+\boldsymbol{Q},l}e^{i(\bar{\boldsymbol{q}}+\boldsymbol{Q})\cdot\boldsymbol{r}}. \tag{110}$$

The complete continuum $\boldsymbol{W}$ field consists of components with different wavevectors and layers: $\boldsymbol{W}(\boldsymbol{r},t) = \sum_{\boldsymbol{Q}l} \boldsymbol{W}_{\bar{\boldsymbol{q}}+\boldsymbol{Q},l}e^{i(\bar{\boldsymbol{q}}+\boldsymbol{Q})\cdot\boldsymbol{r}}$, so does the continuum $\boldsymbol{P}$ field:

$$\boldsymbol{P}(\boldsymbol{r},t) = \frac{1}{\Omega_0}\sum_{l\alpha} Z_\alpha e\boldsymbol{u}_{l\alpha}(\boldsymbol{r},t) = \gamma\sum_{\boldsymbol{Q}l}\boldsymbol{W}_{\bar{\boldsymbol{q}}+\boldsymbol{Q},l}e^{i(\bar{\boldsymbol{q}}+\boldsymbol{Q})\cdot\boldsymbol{r}} = \sum_{\boldsymbol{Q}}\boldsymbol{P}_{\bar{\boldsymbol{q}}+\boldsymbol{Q}}e^{i(\bar{\boldsymbol{q}}+\boldsymbol{Q})\cdot\boldsymbol{r}}, \tag{111}$$

where $\gamma = Z_N e(M_N^{-1}+M_B^{-1})^{1/2}\Omega_0^{-1/2}$ takes the same form as the monolayer case in Eq. (58). We see that the $\boldsymbol{Q}$-component of $\boldsymbol{P}$ and $\boldsymbol{W}$ are related simply through

$$\boldsymbol{P}_{\bar{\boldsymbol{q}}+\boldsymbol{Q}} = \gamma(\boldsymbol{W}_{\bar{\boldsymbol{q}}+\boldsymbol{Q},1} + \boldsymbol{W}_{\bar{\boldsymbol{q}}+\boldsymbol{Q},2}). \tag{112}$$

With the multi-component electric field $\boldsymbol{E}(\boldsymbol{r},t) = \sum_{\boldsymbol{Q}}\boldsymbol{E}_{\bar{\boldsymbol{q}}+\boldsymbol{Q}}e^{i(\bar{\boldsymbol{q}}+\boldsymbol{Q})\cdot\boldsymbol{r}}$, the equation of motion for $\boldsymbol{W}$ becomes a hybrid matrix equation

$$\ddot{W}_{\bar{\boldsymbol{q}}+\boldsymbol{Q},l\mu} = -\sum_{\boldsymbol{Q}'l'\nu}\mathcal{D}_{\boldsymbol{Q}l\mu,\boldsymbol{Q}'l'\nu}(\bar{\boldsymbol{q}})W_{\bar{\boldsymbol{q}}+\boldsymbol{Q}',l'\nu} + \gamma E_{\bar{\boldsymbol{q}}+\boldsymbol{Q},\mu}, \tag{113}$$

where $\mathcal{D}(\bar{\boldsymbol{q}})$ is simply the dynamical matrix in the continuum model of moiré phonons, under the basis in Eq. (99), i.e., Eqs. (101), (102), and (104). For further simplification, we can even neglect the $\bar{\boldsymbol{q}}$-dependence of the force field, i.e., $\mathcal{D}(\bar{\boldsymbol{q}}) = \mathcal{D}(\bar{\boldsymbol{0}})$. If so, the $\bar{\boldsymbol{q}}$-dependence of the PhP only comes from the EM waves, i.e., through the introduction of parameters $\lambda_{\boldsymbol{Q}}^2 = (\bar{\boldsymbol{q}}+\boldsymbol{Q})^2 - \omega^2/c^2$. For a more accurate calculation, we should retain the $\bar{\boldsymbol{q}}$-dependence of the force field.

The following discussion is parallel to the toy model. Suppose $\boldsymbol{w}_{\bar{\boldsymbol{q}}b}$ diagonalizes $\mathcal{D}(\bar{\boldsymbol{q}})$, and the plane waves are expanded using normal modes as $W_{\bar{\boldsymbol{q}}+\boldsymbol{Q},l\mu} = \sum_b U_{\boldsymbol{Q}l\mu,b}(\bar{\boldsymbol{q}})w_{\bar{\boldsymbol{q}},b}$ [notice that $\mathcal{D}(\bar{\boldsymbol{q}})U(\bar{\boldsymbol{q}}) = U(\bar{\boldsymbol{q}})\mathrm{Diag}(\omega_{\bar{\boldsymbol{q}}b}^2)$]. Then, if the system is driven by an electric field at frequency ω, we can solve

$$w_{\bar{\boldsymbol{q}}b} = \frac{\gamma}{\omega_{\bar{\boldsymbol{q}}b}^2 - \omega^2}\sum_{\boldsymbol{Q}l\mu}E_{\bar{\boldsymbol{q}}+\boldsymbol{Q},\mu}U^*_{\boldsymbol{Q}l\mu,b}(\bar{\boldsymbol{q}}). \tag{114}$$

So the polarization

$$P_{\bar{\boldsymbol{q}}+\boldsymbol{Q},\mu} = \gamma\sum_l W_{\bar{\boldsymbol{q}}+\boldsymbol{Q},l\mu} = \gamma\sum_{lb}w_{\bar{\boldsymbol{q}}b}U_{\boldsymbol{Q}l\mu,b}(\bar{\boldsymbol{q}}) = \sum_{\boldsymbol{Q}'\nu}\sum_{bll'}\frac{\gamma^2}{\omega_{\bar{\boldsymbol{q}}b}^2-\omega^2}U_{\boldsymbol{Q}l\mu,b}(\bar{\boldsymbol{q}})U^*_{\boldsymbol{Q}'l'\nu,b}(\bar{\boldsymbol{q}})E_{\bar{\boldsymbol{q}}+\boldsymbol{Q}',\nu}, \tag{115}$$

from which we read

$$\varepsilon_0\Pi_{\mu\nu}^{\boldsymbol{Q}\boldsymbol{Q}'}(\bar{\boldsymbol{q}}) = \gamma^2\sum_{bll'}\frac{U_{\boldsymbol{Q}l\mu,b}(\bar{\boldsymbol{q}})U^*_{\boldsymbol{Q}'l'\nu,b}(\bar{\boldsymbol{q}})}{\omega_{\bar{\boldsymbol{q}}b}^2-\omega^2}. \tag{116}$$

We have listed some results in Fig. S4 calculated using the present continuum model for the 2.65° twisted bilayer hBN. Both the moiré PhP bands and local susceptibility are accurately recovered [compared to those shown in the main text obtained through the lattice model]. We have tried artificially turned off the moiré potential by setting Eq. (108) to zero. In such a case, the system consists of two decoupled monolayers, where

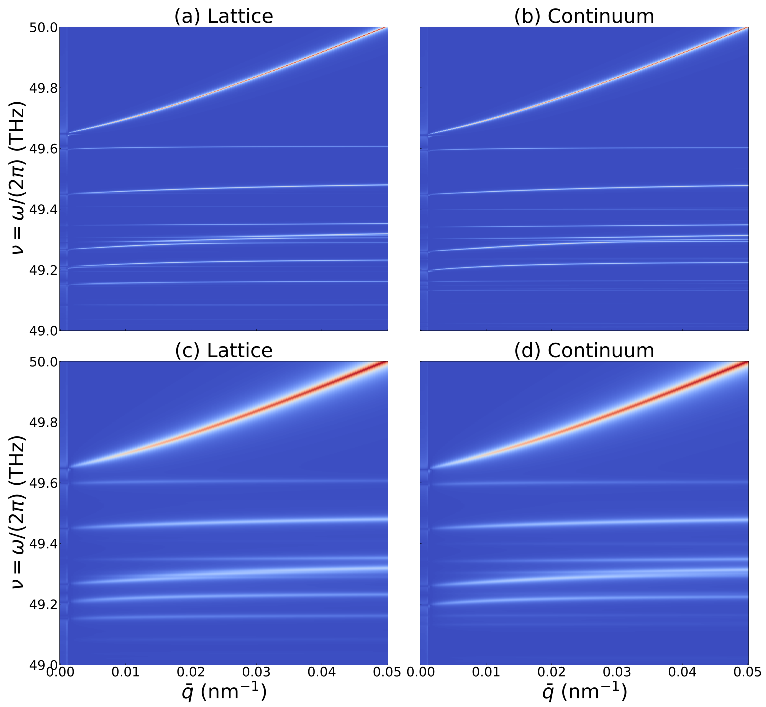

Figure S5: The PhP dispersion of twisted bilayer 2.65° hBN, calculated using (a) the lattice model, $\delta/(2\pi) = 0.001$ THz, (b) the continuum model, $\delta/(2\pi) = 0.001$ THz, (c) the lattice model, $\delta/(2\pi) = 0.01$ THz, and (d) the continuum model, $\delta/(2\pi) = 0.01$ THz. The continuum model is calculated using 37 $\boldsymbol{Q}$ vectors.

all moiré physics disappear: the multiple flat moiré PhP bands are missing, and the local susceptibility shows no signal difference between AA- and AB-stacking points.

To test the accuracy of the continuum model, we plot the PhP dispersion obtained using the lattice and the continuum models in Fig. S5 for comparison. We see that the PhP dispersion above 49.3 THz is well captured by the continuum model. The accuracy of the continuum decreases as the frequency moves away from the critical frequency ω_0, which is a feature of the $k \cdot p$ approximation.

Our next goal is to generalize the continuum model for systems with other twisting angles. At the present stage we have only checked its accuracy for 2.65° hBN, but we expect it to work well over a range of twisting angles. We anticipate that, similar to the BM model [17], the continuum model can only be used when the twisting angle is not too large or too small. For large angles, the continuum approximation itself is not valid. Our continuum model has a intralayer moiré potential that is much stronger than the interlayer part, so they would exhibit strong twisting angle dependence when the angle is vary small (say, $\theta < 0.1°$) where the corrugation effect dominates. In that case, one must be careful to tune the intralayer parameters Eq. (103). The determination of the range of applicability and the model parameters will be left for future research.

5 More details about the moiré response function

5.1 Derivation in quantum case

The response function used in the main text is also valid if phonons are treated quantum mechanically. We rederive it using linear response theory. The displacement operator is

$$\hat{\boldsymbol{u}}(\boldsymbol{r}_{I i\alpha}) = \sum_{\bar{\boldsymbol{q}} b} \sqrt{\frac{\hbar}{2 M_\alpha \omega_{\bar{\boldsymbol{q}} b} N_m}} \boldsymbol{e}_{i\alpha, b}(\bar{\boldsymbol{q}}) e^{i\bar{\boldsymbol{q}}\cdot\boldsymbol{r}_{I i\alpha}} (\hat{a}_{\bar{\boldsymbol{q}} b} + \hat{a}^\dagger_{-\bar{\boldsymbol{q}} b}), \tag{117}$$

where $\hat{a}_{\bar{\boldsymbol{q}}b}$, $\hat{a}^\dagger_{\bar{\boldsymbol{q}}b}$ are operators of phonon mode $\bar{\boldsymbol{q}}, b$, satisfying $[\hat{a}_{\bar{\boldsymbol{q}}b}, \hat{a}^\dagger_{\bar{\boldsymbol{q}}'b'}] = \delta_{\bar{\boldsymbol{q}}\bar{\boldsymbol{q}}'}\delta_{bb'}$.The polarization operator, defined like Eq. (68), is expanded in Fourier space

$$\hat{\boldsymbol{P}}(\boldsymbol{r}) = \sum_{\bar{\boldsymbol{q}}\boldsymbol{Q}} e^{i(\bar{\boldsymbol{q}}+\boldsymbol{Q})\cdot\boldsymbol{r}} \hat{\boldsymbol{P}}_{\bar{\boldsymbol{q}}+\boldsymbol{Q}}, \tag{118}$$

where $\hat{\boldsymbol{P}}_{\bar{\boldsymbol{q}}+\boldsymbol{Q}}$ can be calculated similar to Eq. (76),

$$\hat{\boldsymbol{P}}_{\bar{\boldsymbol{q}}+\boldsymbol{Q}} = \frac{e}{\Omega_m} \sum_b \boldsymbol{S}_{\boldsymbol{Q}b}(\bar{\boldsymbol{q}}) \sqrt{\frac{\hbar}{2\omega_{\bar{\boldsymbol{q}}b} N_m}} (\hat{a}_{\bar{\boldsymbol{q}}b} + \hat{a}^\dagger_{-\bar{\boldsymbol{q}}b}), \tag{119}$$

with the form factor $\boldsymbol{S}_{\boldsymbol{Q}b}(\bar{\boldsymbol{q}})$ defined in Eq. (74).

With an electric field $\boldsymbol{E}_t(\boldsymbol{r}, t) = \sum_{\boldsymbol{Q}} \boldsymbol{E}_{\bar{\boldsymbol{q}}+\boldsymbol{Q},t}(\omega) e^{i(\bar{\boldsymbol{q}}+\boldsymbol{Q})\cdot\boldsymbol{r} - i\omega t}$, the total Hamiltonian is

$$\hat{H} = \hat{H}_0 + \hat{H}_{\text{res}}, \tag{120}$$

where $\hat{H}_0 = \sum_{\bar{\boldsymbol{q}}b} \hbar\omega_{\bar{\boldsymbol{q}}b} \hat{a}^\dagger_{\bar{\boldsymbol{q}}b} \hat{a}_{\bar{\boldsymbol{q}}b}$, and ($\Omega_{\text{tot}} = N_m \Omega_m$)

$$\hat{H}_{\text{res}} = -\int d\boldsymbol{r} \hat{\boldsymbol{P}}(\boldsymbol{r}) \cdot \boldsymbol{E}_t(\boldsymbol{r}, t) = -\Omega_{\text{tot}} \sum_{\boldsymbol{Q}'} \hat{\boldsymbol{P}}_{-\bar{\boldsymbol{q}}-\boldsymbol{Q}'} \cdot \boldsymbol{E}_{\bar{\boldsymbol{q}}+\boldsymbol{Q}',t}(\omega) e^{-i\omega t}. \tag{121}$$

Treating $\hat{H}_{\text{res}}$ as an external coupling, the induced polarization $\boldsymbol{P}_{\bar{\boldsymbol{q}}+\boldsymbol{Q}}(t) = \langle \hat{\boldsymbol{P}}_{\bar{\boldsymbol{q}}+\boldsymbol{Q}} \rangle(t) - \langle \hat{\boldsymbol{P}}_{\bar{\boldsymbol{q}}+\boldsymbol{Q}} \rangle_0$ is given by the Kubo formula as

$$P_{\bar{\boldsymbol{q}}+\boldsymbol{Q},\mu}(t) = \sum_{\boldsymbol{Q}'\nu} \varepsilon_0 \Pi^{\boldsymbol{Q}\boldsymbol{Q}'}_{\mu\nu}(\bar{\boldsymbol{q}}, \omega) E_{\bar{\boldsymbol{q}}+\boldsymbol{Q}',\nu}(\omega) e^{-i\omega t}, \tag{122}$$

where

$$\Pi^{\boldsymbol{Q}\boldsymbol{Q}'}(\bar{\boldsymbol{q}}, \omega) = -\frac{N_m \Omega_m}{\varepsilon_0 \hbar} \sum_{mn} \frac{[\hat{\boldsymbol{P}}_{\bar{\boldsymbol{q}}+\boldsymbol{Q}}]_{mn} [\hat{\boldsymbol{P}}^T_{-\bar{\boldsymbol{q}}-\boldsymbol{Q}'}]_{nm}}{\omega + (E_m - E_n)/\hbar + i0^+} \frac{1}{\mathcal{Z}_0} (e^{-\beta E_m} - e^{-\beta E_n}). \tag{123}$$

Here $\beta = 1/(k_B T)$, $\mathcal{Z}_0 = \text{Tr}(e^{-\beta \hat{H}_0})$ is the partition function, and $[\hat{O}]_{mn} = \langle m|\hat{O}|n\rangle$ is the matrix element in the phonon Fock basis $|m\rangle, |n\rangle$ with energies E_m, E_n, respectively. Since $\hat{P}_{\bar{\boldsymbol{q}}+\boldsymbol{Q}} \propto \hat{a}_{\bar{\boldsymbol{q}}b} + \hat{a}^\dagger_{-\bar{\boldsymbol{q}}b}$, in the summation only the following terms survive

$$|m\rangle = N^{-1/2}_{n,\bar{\boldsymbol{q}}b} \hat{a}_{\bar{\boldsymbol{q}}b} |n\rangle \quad \text{or} \quad |n\rangle = N^{-1/2}_{m,-\bar{\boldsymbol{q}}b} \hat{a}_{-\bar{\boldsymbol{q}}b} |m\rangle, \tag{124}$$

where $N_{n,\bar{\boldsymbol{q}}b}$ is the multiplicity of the $\bar{\boldsymbol{q}}, b$ phonon in state $|n\rangle$. The two cases give $E_n - E_m = \hbar\omega_{\bar{\boldsymbol{q}}b}$ and $E_m - E_n = \hbar\omega_{-\bar{\boldsymbol{q}}b}$, respectively. Using Eq. (132) and the bosonic statistics

$$\mathcal{Z}_0^{-1} \sum_n e^{-\beta E_n} N_{n,\bar{\boldsymbol{q}}b} = (e^{\beta h \omega_{\bar{\boldsymbol{q}}b}} - 1)^{-1}, \tag{125}$$

the calculation follows

$$\begin{aligned}
\Pi^{\boldsymbol{Q}\boldsymbol{Q}'}(\bar{\boldsymbol{q}}, \omega) = &- \frac{e^2}{\varepsilon_0 \Omega_m} \sum_{mn} \sum_{bb'} \frac{\boldsymbol{S}_{\boldsymbol{Q}b}(\bar{\boldsymbol{q}})}{\sqrt{2\omega_{\bar{\boldsymbol{q}}b}}} \frac{\boldsymbol{S}^\dagger_{\boldsymbol{Q}'b'}(\bar{\boldsymbol{q}})}{\sqrt{2\omega_{\bar{\boldsymbol{q}}b'}}} \frac{[\hat{a}_{\bar{\boldsymbol{q}}b} + \hat{a}^\dagger_{-\bar{\boldsymbol{q}}b}]_{mn} [\hat{a}_{-\bar{\boldsymbol{q}}b'} + \hat{a}^\dagger_{\bar{\boldsymbol{q}}b'}]_{nm}}{\omega + (E_m - E_n)/\hbar + i0^+} \frac{1}{\mathcal{Z}_0} (e^{-\beta E_m} - e^{-\beta E_n}) \\
= &\frac{e^2}{\varepsilon_0 \Omega_m} \sum_b \frac{\boldsymbol{S}_{\boldsymbol{Q}b}(\bar{\boldsymbol{q}}) \boldsymbol{S}^\dagger_{\boldsymbol{Q}'b}(\bar{\boldsymbol{q}})}{2\omega_{\bar{\boldsymbol{q}}b}} \left(\frac{1}{\omega - \omega_{\bar{\boldsymbol{q}}b} + i0^+} - \frac{1}{\omega + \omega_{\bar{\boldsymbol{q}}b} + i0^+} \right) (1 - e^{\beta \hbar \omega_{\bar{\boldsymbol{q}}b}}) \sum_n \frac{N_{n,\bar{\boldsymbol{q}}b}}{\mathcal{Z}_0} e^{-\beta E_n} \\
= &\frac{e^2}{\varepsilon_0 \Omega_m} \sum_b \frac{\boldsymbol{S}_{\boldsymbol{Q}b}(\bar{\boldsymbol{q}}) \boldsymbol{S}^\dagger_{\boldsymbol{Q}'b}(\bar{\boldsymbol{q}})}{\omega^2_{\bar{\boldsymbol{q}}b} - \omega^2 - i\omega 0^+}.
\end{aligned} \tag{126}$$

In Ref. [7] the authors derived a moiré-less version of the formula above in the $T = 0$ limit. The derivation here indicates that the expression actually is *temperature-independent*.

5.2 Non-locality and inhomogeneity

The moiré polar system realizes the spatially non-local response, in the sense that

$$\boldsymbol{P}(\boldsymbol{r},t)=\int d\boldsymbol{r}'dt'\varepsilon_0\boldsymbol{\Pi}(\boldsymbol{r},\boldsymbol{r}',t-t')\boldsymbol{E}(\boldsymbol{r}',t'). \tag{127}$$

Here the moiré response function, defined as

$$\boldsymbol{\Pi}(\boldsymbol{r},\boldsymbol{r}',t)=\frac{1}{2\pi\Omega_{\text{tot}}}\int d\omega\sum_{\bar{\boldsymbol{q}}\boldsymbol{Q}\boldsymbol{Q}'}\boldsymbol{\Pi}^{\boldsymbol{Q}\boldsymbol{Q}'}(\bar{\boldsymbol{q}},\omega)e^{i(\bar{\boldsymbol{q}}+\boldsymbol{Q})\cdot\boldsymbol{r}-i(\bar{\boldsymbol{q}}+\boldsymbol{Q}')\cdot\boldsymbol{r}'}e^{-i\omega t}, \tag{128}$$

is invariant under translations with moiré period (not the atomic cell period)

$$\boldsymbol{\Pi}(\boldsymbol{r},\boldsymbol{r}',t)=\boldsymbol{\Pi}(\boldsymbol{r}+\boldsymbol{L}_I,\boldsymbol{r}'+\boldsymbol{L}_I,t). \tag{129}$$

For a general vector $\boldsymbol{a}$ that is incommensurate with the moiré lattice, the non-locality indicates $\boldsymbol{\Pi}(\boldsymbol{r},\boldsymbol{r}',t)\neq\boldsymbol{\Pi}(\boldsymbol{r}+\boldsymbol{a},\boldsymbol{r}'+\boldsymbol{a},t)$, which is different from the moiré-less case. By transforming the above formula into frequency space and setting $\boldsymbol{r}'=\boldsymbol{r}$, we obtain the local susceptibility Eq. (137) discussed in the main text.

5.3 Symmetry properties

For simplicity let us consider the non-degenerate case, i.e., $\omega_{\bar{\boldsymbol{q}}b}\neq\omega_{\bar{\boldsymbol{q}}b'}$ when $b\neq b'$. The time reversal requires

$$\omega_{-\bar{\boldsymbol{q}},b}=\omega_{\bar{\boldsymbol{q}}b},\quad \boldsymbol{e}_{i\alpha,b}(-\bar{\boldsymbol{q}})=\boldsymbol{e}^*_{i\alpha,b}(\bar{\boldsymbol{q}}), \tag{130}$$

while for a point group rotation g of the system, it requires

$$\omega_{g\bar{\boldsymbol{q}},b}=\omega_{\bar{\boldsymbol{q}}b},\quad g\boldsymbol{e}_{g^{-1}(i\alpha),b}(\bar{\boldsymbol{q}})=\boldsymbol{e}_{i\alpha,b}(g\bar{\boldsymbol{q}}). \tag{131}$$

These give the following constraints on the S matrix

$$\boldsymbol{S}_{\boldsymbol{Q}b}(\bar{\boldsymbol{q}})=[\boldsymbol{S}_{-\boldsymbol{Q}b}(-\bar{\boldsymbol{q}})]^*, \tag{132}$$

$$\boldsymbol{S}_{\boldsymbol{Q}b}(\bar{\boldsymbol{q}})=g^{-1}\boldsymbol{S}_{g\boldsymbol{Q},b}(g\bar{\boldsymbol{q}}). \tag{133}$$

As a result, the response function satisfies

$$\Pi^{\boldsymbol{Q}\boldsymbol{Q}'}(\bar{\boldsymbol{q}},\omega)=[\Pi^{-\boldsymbol{Q},-\boldsymbol{Q}'}(-\bar{\boldsymbol{q}},-\omega)]^*, \tag{134}$$

$$\Pi^{\boldsymbol{Q}\boldsymbol{Q}'}(\bar{\boldsymbol{q}},\omega)=g^{-1}\Pi^{g\boldsymbol{Q},g\boldsymbol{Q}'}(g\bar{\boldsymbol{q}},\omega)g. \tag{135}$$

The last identity also holds if there exists degeneracy [in this case we have $g\boldsymbol{S}_{\boldsymbol{Q}b_j}(\bar{\boldsymbol{q}})=\sum_{j'}\boldsymbol{S}_{g\boldsymbol{Q},b_{j'}}(g\bar{\boldsymbol{q}})U^g_{j'j}(\bar{\boldsymbol{q}})$ instead, where $b_{j(j')}$ runs over the degenerate subspace, and the matrix $U^g(\bar{\boldsymbol{q}})$ is unitary]. Besides, the form of Eq. (78) itself has an additional property

$$\Pi^{\boldsymbol{Q}\boldsymbol{Q}'}(\bar{\boldsymbol{q}},\omega)=[\Pi^{\boldsymbol{Q}'\boldsymbol{Q}}(\bar{\boldsymbol{q}},-\omega)]^\dagger. \tag{136}$$

If $\boldsymbol{E}(\boldsymbol{r},t)$ is an eigenmode, then so is $\boldsymbol{E}^*(\boldsymbol{r},t)$, as implied by Eq. (134). This guarantees that the eigenfields can always taken to be real. On the other hand, Eq. (135) indicates that the rotated field $g\boldsymbol{E}(g^{-1}\boldsymbol{r},t)$ is also an eigen solution with the same dispersion.

We now examine the symmetry properties of the local response function $\Pi(\boldsymbol{r},\boldsymbol{r},\omega)$, which is defined as

$$\Pi(\boldsymbol{r},\boldsymbol{r},\omega)=\frac{1}{\Omega_{\text{tot}}}\sum_{\bar{\boldsymbol{q}}\boldsymbol{Q}\boldsymbol{Q}'}\Pi^{\boldsymbol{Q}\boldsymbol{Q}'}(\bar{\boldsymbol{q}},\omega)e^{i(\boldsymbol{Q}-\boldsymbol{Q}')\cdot\boldsymbol{r}}. \tag{137}$$

First, Eq. (136) and the time reversal symmetry Eq. (134) lead to $\Pi^{\boldsymbol{Q}\boldsymbol{Q}'}(\bar{\boldsymbol{q}},\omega)=[\Pi^{-\boldsymbol{Q}',-\boldsymbol{Q}}(-\bar{\boldsymbol{q}},\omega)]^T$, so

$$\Pi(\boldsymbol{r},\boldsymbol{r},\omega)=\frac{1}{\Omega_{\text{tot}}}\sum_{\bar{\boldsymbol{q}}\boldsymbol{Q}\boldsymbol{Q}'}[\Pi^{-\boldsymbol{Q}',-\boldsymbol{Q}}(-\bar{\boldsymbol{q}},\omega)]^Te^{i(\boldsymbol{Q}-\boldsymbol{Q}')\cdot\boldsymbol{r}}=\Pi^T(\boldsymbol{r},\boldsymbol{r},\omega), \tag{138}$$

i.e., the local response matrix is symmetric. Besides, using the property Eq. (135), we have

$$\begin{aligned} g\Pi(\boldsymbol{r},\boldsymbol{r},\omega)g^{-1} =&\frac{1}{\Omega_{\text{tot}}}\sum_{\bar{\boldsymbol{q}}\boldsymbol{Q}\boldsymbol{Q}'} g\Pi^{\boldsymbol{Q}\boldsymbol{Q}}(\bar{\boldsymbol{q}},\omega)g^{-1}e^{i(\boldsymbol{Q}-\boldsymbol{Q}')\cdot\boldsymbol{r}} = \frac{1}{\Omega_{\text{tot}}}\sum_{\bar{\boldsymbol{q}}\boldsymbol{Q}\boldsymbol{Q}'} \Pi^{g\boldsymbol{Q},g\boldsymbol{Q}}(g\bar{\boldsymbol{q}},\omega)e^{ig(\boldsymbol{Q}-\boldsymbol{Q}')\cdot g\boldsymbol{r}} \\ =&\frac{1}{\Omega_{\text{tot}}}\sum_{\bar{\boldsymbol{q}}\boldsymbol{Q}\boldsymbol{Q}'} \Pi^{\boldsymbol{Q}\boldsymbol{Q}}(\bar{\boldsymbol{q}},\omega)e^{i(\boldsymbol{Q}-\boldsymbol{Q}')\cdot g\boldsymbol{r}} = \Pi(g\boldsymbol{r},g\boldsymbol{r},\omega). \end{aligned} \tag{139}$$

For a g-invariant point $\boldsymbol{r}_0$, i.e., there exists a moiré lattice vector $\boldsymbol{L}$ so that $g\boldsymbol{r}_0 = \boldsymbol{r}_0 + \boldsymbol{L}$. Using the periodic property Eq. (129), we get $g\Pi(\boldsymbol{r}_0,\boldsymbol{r}_0,\omega)g^{-1} = \Pi(\boldsymbol{r}_0,\boldsymbol{r}_0,\omega)$. In other words, the local response matrix at a g-invariant point commutes with g.

For both twisted bilayer hBN and $MoTe_2$, $g = C_{3z}$ is a lattice symmetry. Now consider $\boldsymbol{r} = \boldsymbol{r}_{AA}$ and $\boldsymbol{r}_{AB}$. Both are C_{3z}-invariant points: $C_{3z}\boldsymbol{r}_{AA} = \boldsymbol{r}_{AA}$ and $C_{3z}\boldsymbol{r}_{AB} = \boldsymbol{r}_{AB} - \boldsymbol{L}_1^m$ [Fig. S1 (a)]. Therefore, we immediately find $C_{3z}\Pi^{AA(AB)}(\omega)C_{3z}^{-1} = \Pi^{AA(AB)}(\omega)$, where $\Pi^{AA(AB)}(\omega) = \Pi(\boldsymbol{r}_{AA(AB)},\boldsymbol{r}_{AA(AB)},\omega)$. On the other hand, due to Eq. (138), $\Pi^{AA(AB)}$ should be symmetric. As a result, $\Pi^{AA(AB)}$ must be diagonal and, in fact, proportional to the identity matrix:

$$\Pi^{AA(AB)}(\omega) = \Pi^{AA(AB)}_{xx}(\omega)I_{2\times 2}. \tag{140}$$

The above discussion can be generalized. As long as the lattice has an in-plane rotation symmetry $g \neq C_{2z}$, the local response matrix at a g-invariant point is proportional to identity. This is because all 2×2 matrices commuting with a rotation $g \neq C_{2z}$ take the form $AI_{2\times 2} + B\sigma_y$ (σ_y is the Pauli matrix, A, B are complex numbers), and the symmetric property requires $B = 0$. If the lattice has no in-plane rotation symmetry, or has only C_{2z} symmetry (which is absent in polar systems, as otherwise the polarization in Eq. (68) would vanish), then at any point the local response matrix is in general not proportional to identity.

5.4 Representation in moiré-less basis

In the presence of the moiré potential, the eigenvector $\boldsymbol{e}_{i\alpha,b}(\bar{\boldsymbol{q}})$ (with eigenfrequency $\omega_{\bar{\boldsymbol{q}}b}$) is related to the (folded) moiré-less eigenvectors $\boldsymbol{e}_{\alpha,la}(\bar{\boldsymbol{q}}+\boldsymbol{Q})$ (with frequency $\omega^0_{\bar{\boldsymbol{q}}+\boldsymbol{Q},la}$) by Eq. (96) [10, 11]

$$\boldsymbol{e}_{i\alpha,b}(\bar{\boldsymbol{q}}) = \sum_{\boldsymbol{Q}la} \frac{e^{i\boldsymbol{Q}\cdot(\boldsymbol{R}_i+\boldsymbol{\tau}_\alpha)}}{\sqrt{N_a}} \boldsymbol{e}_{\alpha,la}(\bar{\boldsymbol{q}}+\boldsymbol{Q})U_{\boldsymbol{Q}la,b}(\bar{\boldsymbol{q}}). \tag{141}$$

The matrix U is determined by the moiré potential (dynamical matrix) $D^m(\bar{\boldsymbol{q}})$, satisfying $U^\dagger U = UU^\dagger = I$ and $U_{-\boldsymbol{Q}la,b}(-\bar{\boldsymbol{q}}) = U^*_{\boldsymbol{Q}la,b}(\bar{\boldsymbol{q}})$. Using the identity [11]

$$\frac{1}{N_a}\sum_i e^{i\boldsymbol{Q}\cdot\boldsymbol{R}_i} = \delta_{\boldsymbol{Q}\boldsymbol{0}}, \tag{142}$$

the moiré S matrix Eq. (74) can be related to the moiré-less one Eq. (40) through

$$\boldsymbol{S}_{\boldsymbol{Q}b}(\bar{\boldsymbol{q}}) = \sqrt{N_a}\sum_a \boldsymbol{S}_{la}(\bar{\boldsymbol{q}}+\boldsymbol{Q})U_{\boldsymbol{Q}la,b}(\bar{\boldsymbol{q}}). \tag{143}$$

So from Eq. (78) we get

$$\varepsilon_0\boldsymbol{\Pi}^{\boldsymbol{Q}\boldsymbol{Q}'}(\bar{\boldsymbol{q}},\omega) = \frac{e^2}{\Omega_0}\sum_b\sum_{la}\sum_{l'a'} \frac{\boldsymbol{S}_{la}(\bar{\boldsymbol{q}}+\boldsymbol{Q})\boldsymbol{S}^\dagger_{l'a'}(\bar{\boldsymbol{q}}+\boldsymbol{Q}')}{\omega^2_{\bar{\boldsymbol{q}}b}-\omega^2} U_{\boldsymbol{Q}la,b}(\bar{\boldsymbol{q}})U^*_{\boldsymbol{Q}'l'a',b}(\bar{\boldsymbol{q}}), \tag{144}$$

which is equivalent to Eq. (116) if one approximates $\boldsymbol{S}_{la}(\bar{\boldsymbol{q}}+\boldsymbol{Q}) \approx \boldsymbol{S}_{la}(\boldsymbol{0})$. In the moiré-less case where $\boldsymbol{Q}$ is a good quantum number, $U_{\boldsymbol{Q}la,b} = \delta_{\boldsymbol{Q}la,b}$, and $\Pi(\omega)$ reduces to the diagonal moiré-less result of Eq. (44). We see that the off-diagonal elements of $\Pi^{\boldsymbol{Q}\boldsymbol{Q}'}$ arise from the off-diagonal elements of U. From perturbation theory, their strength is proportional to the moiré potential ($\boldsymbol{Q}la \neq \boldsymbol{Q}'l'a'$, $\delta\mathcal{D}$ is the moiré potential):

$$U_{\boldsymbol{Q}la,\boldsymbol{Q}'l'a'}(\bar{\boldsymbol{q}}) \sim \frac{\boldsymbol{e}^\dagger_{la}(\bar{\boldsymbol{q}}+\boldsymbol{Q})\delta\mathcal{D}(\bar{\boldsymbol{q}})\boldsymbol{e}_{l'a'}(\bar{\boldsymbol{q}}+\boldsymbol{Q}')}{\omega^2_{\bar{\boldsymbol{q}}+\boldsymbol{Q}',l'a'} - \omega^2_{\bar{\boldsymbol{q}}+\boldsymbol{Q},la}}. \tag{145}$$

6 Interatomic force constants

We use the frozen phonon method to compute the interatomic force constants (FC), defined as

$$\Phi_{i\alpha\mu,j\beta\nu}(\boldsymbol{r}_{Ii\alpha} - \boldsymbol{r}_{Jj\beta}) = \frac{\partial^2 U}{\partial u_\mu(\boldsymbol{r}_{Ii\alpha})\partial u_\nu(\boldsymbol{r}_{Jj\beta})}. \tag{146}$$

The potential energy $U = U_{\text{intra}} + U_{\text{inter}}$ includes contributions from intralayer and interlayer atomic interactions. Specifically, for hBN, U_{intra} is modeled by the Tersoff potential [18], and U_{inter} is modeled by the registry-dependent interlayer potential [19] tailored for the twisted bilayer hBN [20, 21]. For $MoTe_2$, we use Stillinger-Weber potential [22] parameterized by Jiang [23] to model the intralayer interactions. For the interlayer interactions, parameters fitted for TMD systems [24, 25] are used.

Before computing the FCs, the conjugate-gradient and *fire* minimization algorithms are sequentially performed using the large-scale atomic/molecular massively parallel simulator (LAMMPS) [26] to optimize the simulation cell and atomic positions. We have intentionally avoided performing the non-analytical correction on the dynamical matrix, because otherwise it results in a double counting of the Coulomb force. We note that different FCs computed using different force fields could indeed quantitatively influence the polariton dispersion, since the moiré physics occurs on tiny energy scales. Nevertheless, the qualitative moiré structure of the PhP dispersion should be robust and will not be altered by quantitative differences.

References

[1] Thibault Sohier, Marco Gibertini, Matteo Calandra, Francesco Mauri, and Nicola Marzari. Breakdown of optical phonons' splitting in two-dimensional materials. *Nano Letters*, 17(6):3758–3763, 2017. doi: 10.1021/acs.nanolett.7b01090. URL `https://doi.org/10.1021/acs.nanolett.7b01090`.

[2] Nicholas Rivera, Thomas Christensen, and Prineha Narang. Phonon polaritonics in two-dimensional materials. *Nano Letters*, 19(4):2653–2660, 2019. doi: 10.1021/acs.nanolett.9b00518. URL `https://doi.org/10.1021/acs.nanolett.9b00518`.

[3] Jiade Li, Li Wang, Yani Wang, Zhiyu Tao, Weiliang Zhong, Zhibin Su, Siwei Xue, Guangyao Miao, Weihua Wang, Hailin Peng, Jiandong Guo, and Xuetao Zhu. Observation of the nonanalytic behavior of optical phonons in monolayer hexagonal boron nitride. *Nature Communications*, 15(1):1938, Mar 2024. ISSN 2041-1723. doi: 10.1038/s41467-024-46229-4. URL `https://doi.org/10.1038/s41467-024-46229-4`.

[4] Dominik M. Juraschek and Prineha Narang. Highly confined phonon polaritons in monolayers of perovskite oxides. *Nano Letters*, 21(12):5098–5104, 2021. doi: 10.1021/acs.nanolett.1c01002. URL `https://doi.org/10.1021/acs.nanolett.1c01002`.

[5] Paulo André Dias Gonçalves and Nuno MR Peres. *An introduction to graphene plasmonics*. World Scientific, 2016.

[6] Max Born and Kun Huang. *Dynamical Theory Of Crystal Lattices*. Oxford University Press, 08 1996. ISBN 9780192670083. doi: 10.1093/oso/9780192670083.001.0001. URL `https://doi.org/10.1093/oso/9780192670083.001.0001`.

[7] Nicholas Rivera, Jennifer Coulter, Thomas Christensen, and Prineha Narang. Ab initio calculation of phonon polaritons in silicon carbide and boron nitride, 2018. URL `https://arxiv.org/abs/1809.00058`.

[8] Thibault Sohier, Matteo Calandra, and Francesco Mauri. Two-dimensional fröhlich interaction in transition-metal dichalcogenide monolayers: Theoretical modeling and first-principles calculations. *Phys. Rev. B*, 94:085415, Aug 2016. doi: 10.1103/PhysRevB.94.085415. URL `https://link.aps.org/doi/10.1103/PhysRevB.94.085415`.

[9] Guangwei Hu, Qingdong Ou, Guangyuan Si, Yingjie Wu, Jing Wu, Zhigao Dai, Alex Krasnok, Yarden Mazor, Qing Zhang, Qiaoliang Bao, Cheng-Wei Qiu, and Andrea Alù. Topological polaritons and photonic magic angles in twisted α-moo3 bilayers. *Nature*, 582(7811):209–213, Jun 2020. ISSN 1476-4687. doi: 10.1038/s41586-020-2359-9. URL `https://doi.org/10.1038/s41586-020-2359-9`.

[10] Wangqian Miao, Chu Li, Xu Han, Ding Pan, and Xi Dai. Truncated atomic plane wave method for subband structure calculations of moiré systems. *Phys. Rev. B*, 107:125112, Mar 2023. doi: 10.1103/PhysRevB.107.125112. URL https://link.aps.org/doi/10.1103/PhysRevB.107.125112.

[11] Hao Shi, Wangqian Miao, and Xi Dai. Moiré optical phonons coupled to heavy electrons in magic-angle twisted bilayer graphene. *Phys. Rev. B*, 111:155126, 4 2025. doi: 10.1103/PhysRevB.111.155126. URL https://link.aps.org/doi/10.1103/PhysRevB.111.155126.

[12] Michał Papaj and Cyprian Lewandowski. Probing correlated states with plasmons. *Science Advances*, 9 (17):eadg3262, 2023. doi: 10.1126/sciadv.adg3262. URL https://www.science.org/doi/abs/10.1126/sciadv.adg3262.

[13] Ramon Cuscó, Luis Artús, James H. Edgar, Song Liu, Guillaume Cassabois, and Bernard Gil. Isotopic effects on phonon anharmonicity in layered van der waals crystals: Isotopically pure hexagonal boron nitride. *Phys. Rev. B*, 97:155435, Apr 2018. doi: 10.1103/PhysRevB.97.155435. URL https://link.aps.org/doi/10.1103/PhysRevB.97.155435.

[14] Alexander J. Giles, Siyuan Dai, Igor Vurgaftman, Timothy Hoffman, Song Liu, Lucas Lindsay, Chase T. Ellis, Nathanael Assefa, Ioannis Chatzakis, Thomas L. Reinecke, Joseph G. Tischler, Michael M. Fogler, J. H. Edgar, D. N. Basov, and Joshua D. Caldwell. Ultralow-loss polaritons in isotopically pure boron nitride. *Nature Materials*, 17(2):134–139, Feb 2018. ISSN 1476-4660. doi: 10.1038/nmat5047. URL https://doi.org/10.1038/nmat5047.

[15] Ramon Cuscó, James H. Edgar, Song Liu, Jiahan Li, and Luis Artús. Isotopic disorder: The prevailing mechanism in limiting the phonon lifetime in hexagonal bn. *Phys. Rev. Lett.*, 124:167402, Apr 2020. doi: 10.1103/PhysRevLett.124.167402. URL https://link.aps.org/doi/10.1103/PhysRevLett.124.167402.

[16] Long Zhang, Fengcheng Wu, Shaocong Hou, Zhe Zhang, Yu-Hsun Chou, Kenji Watanabe, Takashi Taniguchi, Stephen R. Forrest, and Hui Deng. Van der waals heterostructure polaritons with moiré-induced nonlinearity. *Nature*, 591(7848):61–65, Mar 2021. ISSN 1476-4687. doi: 10.1038/s41586-021-03228-5. URL https://doi.org/10.1038/s41586-021-03228-5.

[17] Rafi Bistritzer and Allan H. MacDonald. Moiré bands in twisted double-layer graphene. *Proceedings of the National Academy of Sciences*, 108(30):12233–12237, 2011. doi: 10.1073/pnas.1108174108. URL https://www.pnas.org/doi/abs/10.1073/pnas.1108174108.

[18] J. Tersoff. New empirical approach for the structure and energy of covalent systems. *Phys. Rev. B*, 37:6991–7000, Apr 1988. doi: 10.1103/PhysRevB.37.6991. URL https://link.aps.org/doi/10.1103/PhysRevB.37.6991.

[19] Aleksey N. Kolmogorov and Vincent H. Crespi. Registry-dependent interlayer potential for graphitic systems. *Phys. Rev. B*, 71:235415, Jun 2005. doi: 10.1103/PhysRevB.71.235415. URL https://link.aps.org/doi/10.1103/PhysRevB.71.235415.

[20] Wengen Ouyang, Davide Mandelli, Michael Urbakh, and Oded Hod. Nanoserpents: Graphene nanoribbon motion on two-dimensional hexagonal materials. *Nano Letters*, 18(9):6009–6016, 2018. doi: 10.1021/acs.nanolett.8b02848. URL https://doi.org/10.1021/acs.nanolett.8b02848.

[21] Wengen Ouyang, Ido Azuri, Davide Mandelli, Alexandre Tkatchenko, Leeor Kronik, Michael Urbakh, and Oded Hod. Mechanical and tribological properties of layered materials under high pressure: Assessing the importance of many-body dispersion effects. *Journal of Chemical Theory and Computation*, 16(1): 666–676, 2020. doi: 10.1021/acs.jctc.9b00908. URL https://doi.org/10.1021/acs.jctc.9b00908.

[22] Frank H. Stillinger and Thomas A. Weber. Computer simulation of local order in condensed phases of silicon. *Phys. Rev. B*, 31:5262–5271, Apr 1985. doi: 10.1103/PhysRevB.31.5262. URL https://link.aps.org/doi/10.1103/PhysRevB.31.5262.

[23] Jin-Wu Jiang. Parametrization of stillinger–weber potential based on valence force field model: application to single-layer mos2 and black phosphorus. *Nanotechnology*, 26(31):315706, jul 2015. doi: 10.1088/0957-4484/26/31/315706. URL https://dx.doi.org/10.1088/0957-4484/26/31/315706.

[24] Wengen Ouyang, Reut Sofer, Xiang Gao, Jan Hermann, Alexandre Tkatchenko, Leeor Kronik, Michael Urbakh, and Oded Hod. Anisotropic interlayer force field for transition metal dichalcogenides: The case of molybdenum disulfide. *Journal of Chemical Theory and Computation*, 17(11):7237–7245, 2021. doi: 10.1021/acs.jctc.1c00782. URL https://doi.org/10.1021/acs.jctc.1c00782.

[25] Wenwu Jiang, Reut Sofer, Xiang Gao, Alexandre Tkatchenko, Leeor Kronik, Wengen Ouyang, Michael Urbakh, and Oded Hod. Anisotropic interlayer force field for group-vi transition metal dichalcogenides. *The Journal of Physical Chemistry A*, 127(46):9820–9830, 2023. doi: 10.1021/acs.jpca.3c04540. URL https://doi.org/10.1021/acs.jpca.3c04540.

[26] Aidan P. Thompson, H. Metin Aktulga, Richard Berger, Dan S. Bolintineanu, W. Michael Brown, Paul S. Crozier, Pieter J. in 't Veld, Axel Kohlmeyer, Stan G. Moore, Trung Dac Nguyen, Ray Shan, Mark J. Stevens, Julien Tranchida, Christian Trott, and Steven J. Plimpton. Lammps - a flexible simulation tool for particle-based materials modeling at the atomic, meso, and continuum scales. *Computer Physics Communications*, 271:108171, 2022. ISSN 0010-4655. doi: https://doi.org/10.1016/j.cpc.2021.108171. URL https://www.sciencedirect.com/science/article/pii/S0010465521002836.
